# Phylogenetic analyses suggest that diversification and body size evolution are independent in insects

**DOI:** 10.1186/s12862-015-0570-3

**Published:** 2016-01-08

**Authors:** James L. Rainford, Michael Hofreiter, Peter J. Mayhew

**Affiliations:** Department of Biology, University of York, Heslington, York, YO10 5DD UK; Institute of Biochemistry and Biology, Faculty of Mathematics and Natural Sciences, University of Potsdam, Karl-Liebknecht-Str. 24-25, 14476 Potsdam, Germany

**Keywords:** Body size, Diversification, Hexapoda, Insects, Phylogeny

## Abstract

**Background:**

Skewed body size distributions and the high relative richness of small-bodied taxa are a fundamental property of a wide range of animal clades. The evolutionary processes responsible for generating these distributions are well described in vertebrate model systems but have yet to be explored in detail for other major terrestrial clades. In this study, we explore the macro-evolutionary patterns of body size variation across families of Hexapoda (insects and their close relatives), using recent advances in phylogenetic understanding, with an aim to investigate the link between size and diversity within this ancient and highly diverse lineage.

**Results:**

The maximum, minimum and mean-log body lengths of hexapod families are all approximately log-normally distributed, consistent with previous studies at lower taxonomic levels, and contrasting with skewed distributions typical of vertebrate groups. After taking phylogeny and within-tip variation into account, we find no evidence for a negative relationship between diversification rate and body size, suggesting decoupling of the forces controlling these two traits. Likelihood-based modeling of the log-mean body size identifies distinct processes operating within Holometabola and Diptera compared with other hexapod groups, consistent with accelerating rates of size evolution within these clades, while as a whole, hexapod body size evolution is found to be dominated by neutral processes including significant phylogenetic conservatism.

**Conclusions:**

Based on our findings we suggest that the use of models derived from well-studied but atypical clades, such as vertebrates may lead to misleading conclusions when applied to other major terrestrial lineages. Our results indicate that within hexapods, and within the limits of current systematic and phylogenetic knowledge, insect diversification is generally unfettered by size-biased macro-evolutionary processes, and that these processes over large timescales tend to converge on apparently neutral evolutionary processes. We also identify limitations on available data within the clade and modeling approaches for the resolution of trees of higher taxa, the resolution of which may collectively enhance our understanding of this key component of terrestrial ecosystems.

**Electronic supplementary material:**

The online version of this article (doi:10.1186/s12862-015-0570-3) contains supplementary material, which is available to authorized users.

## Background

One of the most prevalent patterns observed in natural systems is the overrepresentation of small-bodied taxa [1]. The observation of right skew in body size distributions, following transformation to the log scale, has been made for a variety of vertebrate clades [2–4] and provides the basis for a variety of size-selective diversification mechanisms that have been previously proposed as general models for the macroevolution of animals (reviewed in [1, 4]). Despite widespread interest in these patterns, comparatively little effort has been spent in examining whether such relationships are truly universal and there is limited evidence for their presence across major non-vertebrate lineages [5–7]. In this study, we explore the relationship between species richness and body size, and the universality of size biased diversification, in one of the largest terrestrial invertebrate clades, the six-legged arthropods or Hexapoda.

Interest in body size distributions relates to the importance of size in impacting on an organism’s ecology and thus potential evolution and diversification. Body size determines the scale of an organism’s interactions within the fractal structure of natural environments [8, 9], the relative strength of gravitational (i.e. body weight) vs. viscous and inertial forces [10] and, via surface area to volume ratios and the scaling of exchange networks, controls the rates of metabolic processes such as temperature response [11] and gas diffusion [12]. As a consequence, body size impacts on almost every major life history trait including: growth, parental investment, range size, dispersal and degree of host specificity (see [13–15], and references therein, for reviews of Hexapoda).

Based on these observations a number of size-dependent mechanisms linked to clade diversification have been proposed (reviewed in [3, 4]). These include; hard limits on minimum size, which restrict random character change [16], energetic models emphasizing the relative efficiency of small body sizes in the production of offspring [11, 17], and fractal environmental models, exploring the capacity for small-bodied taxa to more finely subdivide a given environmental landscape [8]. The relationship of these processes to macro-evolutionary diversification remains incompletely understood including, for example, the relative contributions of size-biased cladogenesis (i.e. small taxa being more prone to speciation) [2], directional bias in size evolution within lineages; e.g. “Copes rule” [18], and size-biased extinction [19], on the generation of observed size distributions. Testing the predictions of these models, e.g. the presence of a relationship between clade richness and body size, as well as more generally exploring the processes that may underlie size evolution, requires that we extend our perspectives to encompass other major lineages that may show differences from our vertebrate model systems [20].

The extreme species richness of hexapod clades, which collectively account for over half of all described species, is one of the most well-known features of terrestrial biomes [21]. Hexapoda are also morphologically diverse, including body lengths ranging over four orders of magnitude, comparable with the range of well-studied mammal and bird radiations [13]. The longest known hexapods are females of the phasmid (stick-insect) *Phobaeticus chani* with specimens up to 357 mm long in body length. By contrast, the smallest recognized adult insect, the male of the mymarid wasp *Dicopomorpha echmepterygis* has a total body length of merely 139 μm (or 0.139 mm) [13] (see [22] for further examples of extreme miniaturization in hexapods). Evidence to suggest that processes in hexapod size evolution may be distinct from larger vertebrate groups includes taxonomic compilations (e.g. [23]), regional faunal data (e.g. [24, 25]) and broad-scale continental surveys [26], all of which suggest that compared with vertebrates hexapods exhibit relatively little right skew in the distribution of log body size [13, 15]. Likewise, where formal phylogenetic tests of association between clade richness and body size have been conducted for hexapod sub-clades, they have generally failed to recover evidence for small size promoting richness within the group (e.g. [27]), with one study even identifying the opposite pattern with respect to Anisoptera (dragonflies) [28].

In addition to these apparent divergences from size-structured models there are also potential interactions between size evolution and other hexapod traits, several of which have been previously explored as correlates of species richness including complete metamorphosis, and dietary substrate [21, 29, 30]. Metamorphosis has the potential to structure size evolution via the promotion of modularization of life history stages, and the separation of selection pressures on larval and adult stages [13, 31]. This process is taken to extremes in Holometabola, where during metamorphosis there is a fundamental reorganization of the body plan [32], and as a result various authors have suggested divergent processes of size evolution associated with this clade (it should be noted, however, that the manifestation of these effects in terms of models of trait evolution remains poorly understood [13, 33]).

The recent and growing consensus with regard to hexapod higher taxonomic relationships from molecular markers e.g. [30, 34, 35] provides us, for the first time, with a framework for exploring large scale patterns of trait evolution within the group. In this study, we combine a published phylogeny of insect higher taxa [30] with comprehensive descriptive information regarding size variation within the clade to explore patterns of body size evolution and its relationship with clade diversification. Hypotheses we test include: a) if the apparent lack of skew in body size distributions (on the log scale) identified for regional faunas can be identified in a global phylogenetic perspective on hexapod body size, b) if consistent relationships between clade richness and body size occur after accounting for phylogeny and size variation within terminal groups. In addition, we explore the probable evolutionary process that may underpin size evolution in hexapods, and whether different major clades (e.g. Holometabola or major orders) are associated with divergent evolutionary processes, as has previously demonstrated in mammals [36], with an aim to explore the possible roles of key innovations such as complete metamorphosis [30].

## Results

### Frequency distribution of body sizes

**Fig. 1:**
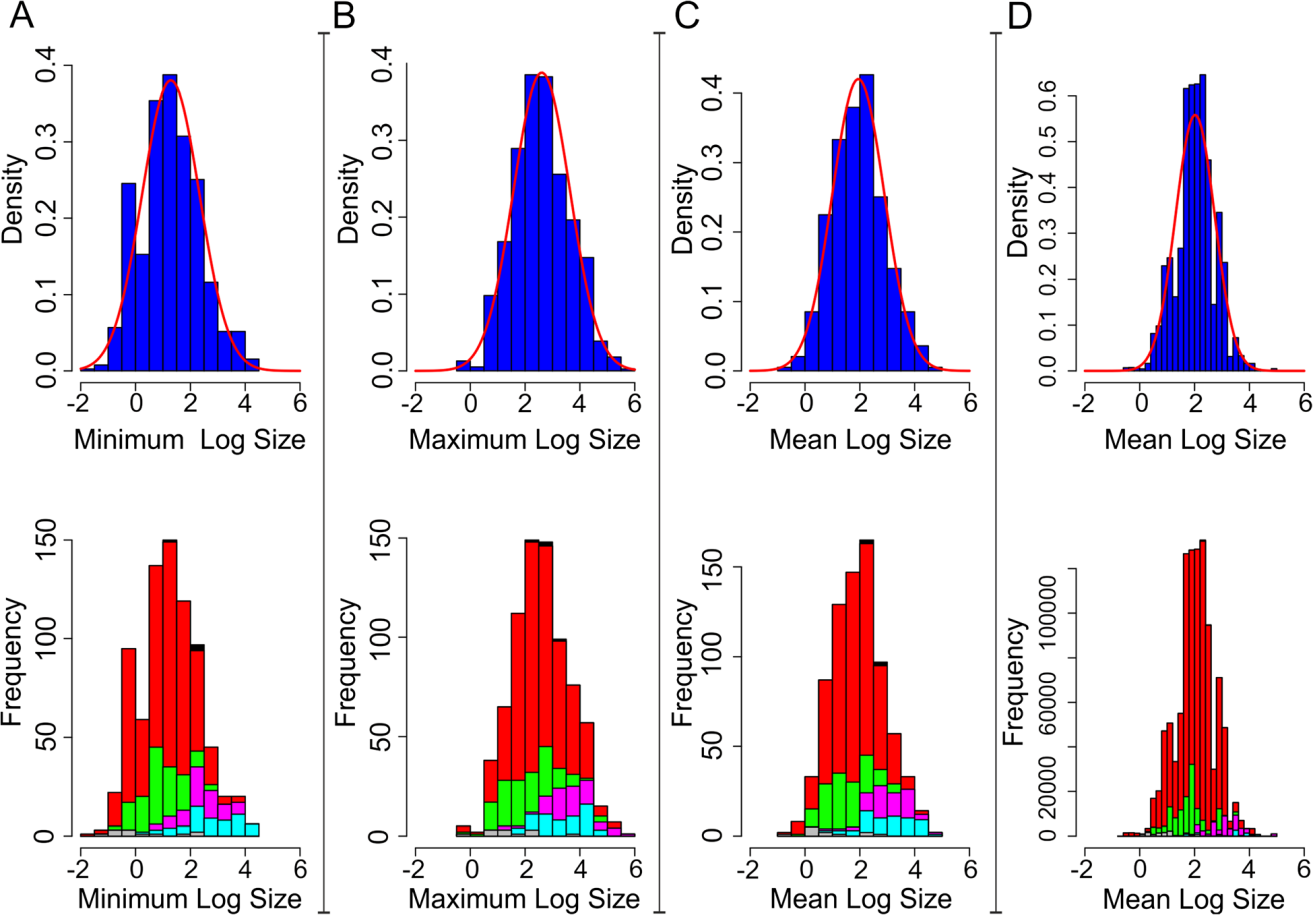
Histograms of raw body length data and estimated mean-of-logs lengths (D; corrected for clade richness). Histograms of **a** Minimum log body size (ln (mm), Skewness = 0.3333) **b** Maximum log body size (ln (mm), Skewness = 0.07517) **c** Calculated mean log body size; for terminal groups used in this analysis (ln (mm), Skewness = 0.2102), **d** Mean size with each terminal group represented proportionally to its richness (ln (mm), Skewness = -0.0285). Curves on upper panels reflect normal distributions with the same mean and standard deviation as the observed data. Colors in lower panels show breakdown of size classes by major taxonomic group; Red - Holometabola, Green - Paraneoptera, Magenta - Polyneoptera, Cyan - Palaeoptera, Black - Basal insects, Grey - Entognatha

Body length range data were gathered for 774 higher taxa of insects (resolved primarily to the family level; Additional file 1: Table S1). The frequency distributions of the observed values of mean-of-logs (mean of the logged values of the size range limits for each higher taxon), log maximum and log minimum body length for terminal taxa are shown in Fig. 1. In all three cases the overall distributions are approximately normal (two-sided Agostino test, log minimum: skew = 0.3333, z = 2.455, *p*-value = 0.0141, log maximum: skew = 0.0752, z = 0.567, *p*-value = 0.5706, mean-of-logs: skew = 0.210, z = 1.572, *p* = 0.116), although the distribution of minimum sizes shows a small secondary peak associated with an over-prevalence of taxa reported as bounded at 1 mm (commonly used for convenience in descriptions of small taxa). When mean values are weighted according to their species richness, the resulting distribution shows a significant skew towards larger body sizes (skew = -0.0290, z = -7.91, p-value = <0.001) running contrary to the expectations of the paradigm described above.

Comparing major clades we can identify pronounced differences in typical size distributions observed among groups. As Holometabola, the most diverse clade (more than 75 % of all extant hexapods) [32] account for the majority of the terminals included in this study (508 out of 775), it is unsurprising that the size distribution of Holometabola (insects with complete metamorphosis) mirrors that of hexapods as a whole, with similar average size to the global mean (Hexapoda; (log) mean = 1.946 ln (mm), sd = 0.9491 ln (mm), Holometabola; (log) mean = 1.8032 ln (mm), sd = 0.8078 ln (mm)). By contrast both the clades Entognatha (non-insect hexapods including springtails; mean =0.8879 ln (mm), sd = 1.061 ln (mm) and Paraneoptera (true bugs and their relatives; mean = 1.5506 ln (mm), sd = 0.7755 ln (mm) are predominantly composed of groups falling at the small end of the size spectrum, the latter particularly with respect to minimum sizes, while large insects include disproportionate representation of Polyneoptera (mean = 3.045 ln (mm), sd = 0.7455 ln (mm)) and Palaeoptera (particularly large bodied Odonata (dragonflies)); mean = 3.060 ln (mm), sd = 0.8825 ln (mm)).

**Fig. 2:**
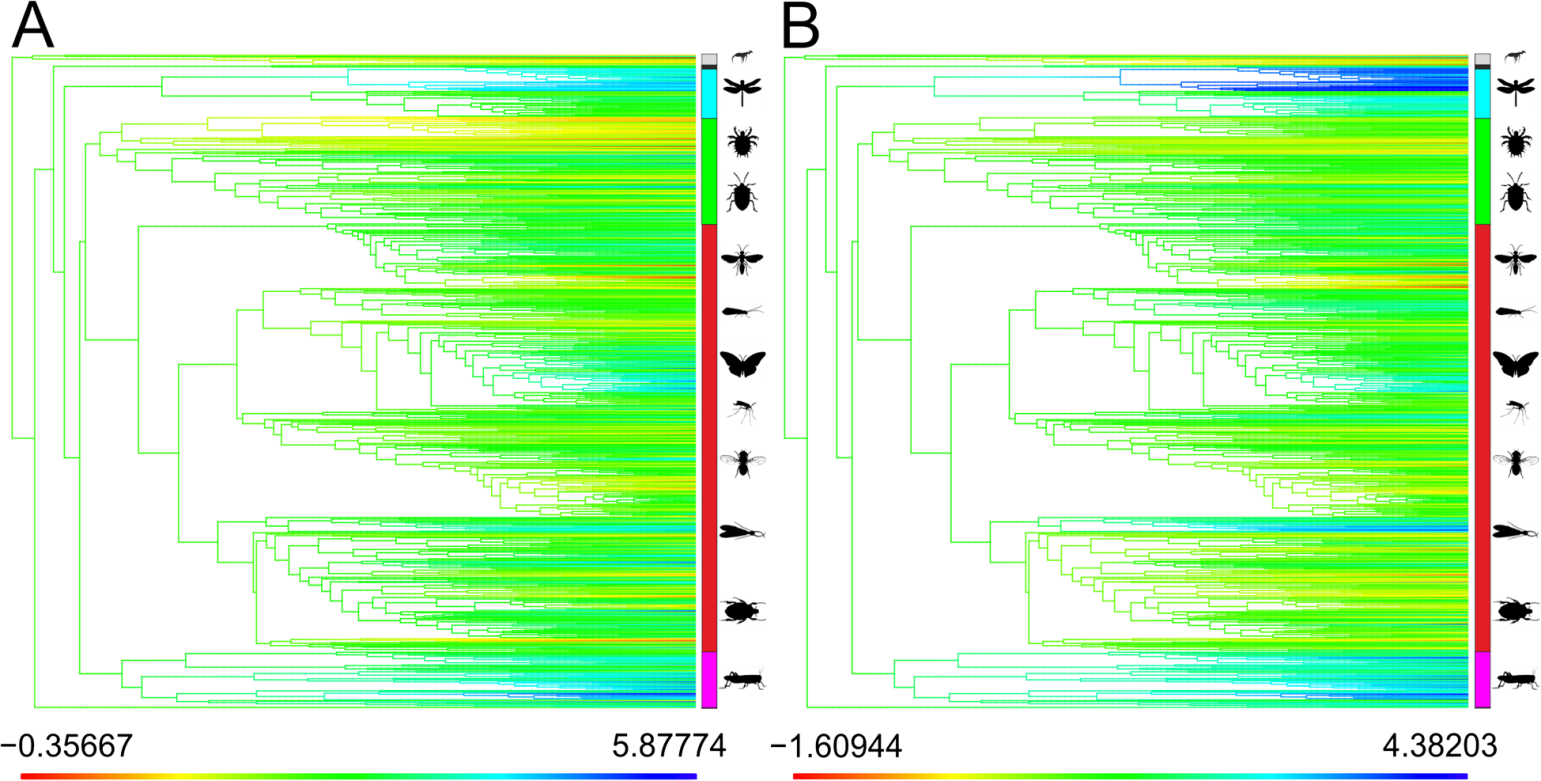
Phylogenetic plot of (log) size traits. **a** mean-of-logs body length; (**b**) estimated standard deviation. Ancestral reconstruction of internal nodes based on a BM process (ancML) (Revel 2013). Lower bars denote the minimum and maximum values of observed traits (ln (mm)); coloration on a red to blue scale. Terminal bars denote membership of major clades; colors as Fig. 1

The value of the inferred standard deviation of the terminal distributions shows a rather different phylogenetic pattern from that of the mean size values, although after taking phylogeny into account the two are strongly correlated (PGLS [37] assuming a Brownian covariance structure: Estimate = 0.4219, SE = 0.1830, t = 2.3049, *p* = 0.0214). Clades associated with particularly low values of standard deviation (implying relatively little size variation after accounting for species richness within terminal groups) include Trichoptera, Neuropterida (lacewings and relatives), Psocodea and Odonata while the largest values occur in Coleoptera and advanced Lepidoptera (Fig. 2), with the single largest value occurring in the morphologically diverse (4-39 mm) but species poor Lepidoptera family *Aididae* (6 species).

### Phylogenetic distribution of body size and ancestral states

The above patterns are reinforced on the phylogenetic ancestral reconstruction plots for the group (Fig. 2, Additional file 1: Figure S1), in which the following clades show strong deviations from the average size dynamics: Odonata (with respect to larger than average minimum body size), Psocodea (booklice and lice; small maximum sizes), micro-hymenoptera (the smallest members of Holometabola with particularly small minimum size bounds) and various polyneopteran clades, notably Phasmatodea and Orthoptera. Beyond these limited examples, the majority of hexapod higher taxa log-means lie close to global average size, and ancestral reconstruction of internal nodes rapidly approaches this value as an approximation of the global ancestral state.

Evidence of phylogenetic signal was recovered in both the full dataset and in all the major sub-clades (Table 1) with very strong support, with the exception of Entognatha, where evidence of structuring is present but support is much lower (likely due to the small number of tips on this subtree: 12). Blomberg’s K values indicate that Hexapoda as a whole demonstrate somewhat lower values of K than would be expected under a Brownian motion (BM) process, consistent with related species resembling one another less than under the expected BM distribution (see further discussion below). Similar patterns are also identified in Holometabola and Polyneoptera. By contrast, Paraneoptera and Palaeoptera show strong tendencies towards higher-than-expected values of K, indicating differences in the size evolution process among major clades.

**Table 1:** Tests of phylogenetic signal within major clades incorporating within-terminal standard error

Taxa	Blomberg’s K	Sigma^2^ rate parameter	Model log likelihood	P randomization test
Hexapoda	0.8870	0.002368	−778.95	<0.001
Holometabola	0.6864	0.002694	−515.43	<0.001
Paraneoptera	1.3166	0.001436	−117.07	<0.001
Polyneoptera	0.8144	0.002122	−66.26	<0.001
Palaeoptera	1.7806	0.001467	−40.192	<0.001
Entognatha	1.1244	0.002574	−15.711	0.0247

### Body size and species richness

**Fig. 3:**
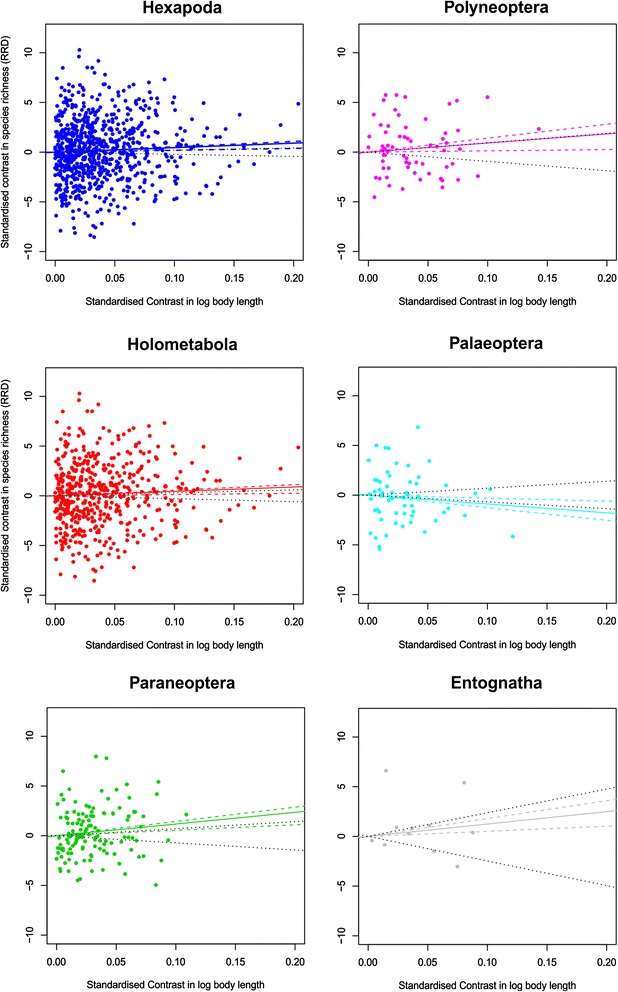
Plots of Standardized contrasts for richness (RDD) and body length (ln (mm)). Solid lines denote the relationship inferred from the mean values in Macrocaic. Dashed colored lines are the 95 % CI based on 50,000 parametric bootstraps taking into account the variance present among terminal groups. Dotted black lines denote the equivalent null intervals calculated on tip randomizations. Statistical information for relationships in Table 3

The standardized contrasts in body size and relative rate difference (RRD; defined as, ln (N_1_/N_2_), where N_1_ = richness of descendant clade with larger body size, and N_2_ = the richness of the other descendant clade [38–40]) across major clades are plotted in Fig. 3. The estimated relationships through the origin were calculated on the observed mean-of-log sizes and confidence intervals were based on the parametric bootstrap samples as drawn from the estimated terminal distributions for both observed (colored) and randomized (black) data (parameter values in Table 2). Overall, the data for Hexapoda support the presence of a weak positive relationship between richness and body size within the clade, although following the parametric bootstrap this relationship is not significant once the uncertainty of terminal states is taken into account. Similar patterns of null relationships once tip variance is taken into consideration occur in all of the major sub-clades examined, although in the case of Palaeoptera the direction of the relationship observed is negative. When these statistics were recalculated based on PDI (Additional file 1: Table S2) no significant relationships were observed between mean size and richness, rendering further parametric bootstrapping redundant.

**Table 2:** Macrocaic analysis of contrasts in RRD and vs. mean-of-logs size for major hexapod clades (Fig. 3)

Taxa	N	Estimate	(Adj) R^2^	SE	t	p	Obs. QR		NULL QR	
							2.5 %	97.5 %	2.5 %	97.5 %
Hexapoda	773	4.538	0.004203	2.219	2.045	0.0412^a^	1.886	5.383	−2.127	2.106
Holometabola	507	4.415	0.003232	2.715	1.626	0.105	1.246	5.580	−2.944	2.969
Non-Holometabola	265	5.416	0.003874	3.801	1.425	0.155	1.927	7.304	−3.159	3.178
Paraneoptera	126	11.759	0.02523	5.696	2.064	0.0411^a^	5.495	14.35	−7.172	7.079
Polyneoptera	64	9.135	0.009866	7.139	1.28	0.205	1.256	14.02	−9.385	9.407
Palaeoptera	57	−8.866	−0.00021	8.919	−0.994	0.325	−12.63	−2.987	−6.800	6.986
Ectognatha	11	12.43	−0.04417	17.00	0.731	0.481	5.118	17.94	−24.74	23.82

Data shown are the results of parametric bootstrap, with 50,000 replicates, Shown are the observed quartile ranges (Obs. QR) and those of the Null tip-randomized data (NULL QR). ^a^ indicates a significant relationship prior to parametric bootstrap (but not after)

### Process of body size evolution

Considering the potential processes responsible for generating observed patterns of size evolution (see Methods), our data suggest that, of our process based models; the majority of hexapod clades favor simple Brownian motion, with the exception of Holometabola, where the favored process is an single stationary peak (SSP/OU) model with convergence on a single global optimum or elevated diversification at distant tips (Table 3, Additional file 1: Table S3). However, when models without an explicit generating process are considered (i.e. lambda and white noise (WN)), this picture changes, such that for Hexapoda as a whole and Holometabola, there is evidence for considerable non-phylogenetic signal in body size, resulting in lambda values that significantly diverge from the expectations of BM (although in all cases the WN model with no phylogenetic signal is strongly rejected, see also Table 1). Similar patterns are obtained when the major holometabolan orders are examined individually, with Hymenoptera (bees, wasps and ants), Coleoptera (beetles) and Lepidoptera (moths and butterflies) all favoring BM processes, while Diptera (flies) shows strong evidence for non-phylogenetic signal (thus favoring the lambda model). The implications of these differences for our understanding of size evolution in hexapods, and particularly within Holometabola and Diptera, will be explored below.

**Table 3:** Parameter estimates and relative likelihoods for models of mean-of-logs body size incorporating within-terminal standard error

Clade	Model	Sigma squared	z0	a/ delta/alpha/lambda	LnLik	k	AICc	Delta AiCc from optimal model	Akaike weights
Hexapoda	BM	0.002403	1.749		−779.4	2	1562.7	21.031	0.00003
	EB	0.002404	1.748	−1e-06*	−779.4	3	1564.7	23.051	0.00001
	delta	0.002196	1.766	1.129	−779.1	3	1564.3	22.627	0.00001
	SSP	0.002666	1.764	0.000591	−778.0	3	1562.1	20.434	0.00004
	**lambda**	**0.001957**	**1.759**	**0.92093**	**−767.8**	**3**	**1541.7**	**0**	**0.9991**
	WN	0.8985	1.946		−1057.3	2	2118.7	576.99	0.0000
Holometabola	BM	0.002726	1.846		−515.4	2	1034.8	17.571	0.0002
	EB	0.002727	1.846	−1e-06*	−515.4	3	1036.9	19.600	0.0001
	delta	0.001787	1.802	1.881	−511.2	3	1028.5	11.265	0.0035
	SSP	0.003613	1.830	0.001923	−510.7	3	1027.4	10.170	0.0061
	**lambda**	**0.002138**	**1.845**	**0.89028**	**−505.6**	**3**	**1017.3**	**0**	**0.9901**
	WN	0.6498	1.803		−611.9	2	1227.8	210.52	0.0000
Paraneoptera	**BM**	**0.001469**	**1.132**		**−117.0**	**2**	**238.2**	**0**	**0.3939**
	EB	0.001518	1.130	−0.000111	−117.0	3	240.3	2.094	0.1382
	delta	0.001559	1.119	0.9031	−117.0	3	240.1	1.9781	0.1465
	SSP	0.001469	1.132	0.00	−117.0	3	240.3	2.0983	0.1379
	lambda	0.001368	1.139	0.9343	−116.7	3	239.7	1.5276	0.1835
	WN	0.5961	1.531		−147.4	2	299.0	60.78	0.0000
Polyneoptera	**BM**	**0.002121**	**2.759**		**−66.26**	**2**	**136.7**	**0.1955**	**0.2922**
	EB	0.002121	2.759	−1e-06*	−66.26	3	138.9	2.3961	0.0972
	delta	0.001389	2.822	2.186	−65.06	3	136.5	0	0.3221
	SSP	0.003247	2.812	0.002286	−65.60	3	137.6	1.081	0.1876
	lambda	0.002005	2.765	0.9636	−66.22	3	138.8	2.334	0.1003
	WN	0.5465	3.045		−72.66	2	149.5	12.99	0.0005
Palaeoptera	**BM**	**0.001485**	**2.918**		**−40.18**	**2**	**84.58**	**0**	**0.3195**
	EB	0.002088	2.917	−0.001169	−40.06	3	86.57	1.991	0.1181
	delta	0.002322	2.938	0.5462	−39.51	3	85.46	0.8857	0.2052
	SSP	0.001485	2.918	0.00	−40.18	3	86.80	2.226	0.1050
	lambda	0.00119	2.928	0.8993	−39.30	3	85.05	0.4729	0.2522
	WN	0.7646	3.060		−74.55	2	153.3	68.73	0.0000
Entognatha	**BM**	**0.002414**	**1.074**		**−15.71**	**2**	**36.75**	**0**	**0.5003**
	EB	0.01257	1.048	−0.006225	−15.16	3	39.31	2.561	0.1390
	delta	0.002921	1.070	0.6378	−15.58	3	40.16	3.407	0.0911
	SSP	0.002414	1.074	0.00	−15.71	3	40.42	3.667	0.0800
	lambda	0.002414	1.074	1	−15.71	3	40.42	3.667	0.0800
	WN	1.0335	0.888		−17.23	2	39.79	3.035	0.1097

Models and relevant parameters are denoted as follows: BM: Brownian motion (Sigma squared: ML estimate of rate of the underlying size evolution, z0: ML estimate of value for the root state); EB: Early burst model (a: exponential rate scale for relationship through time); Delta: Pagel’s delta rate change through time model (delta: tree scaling parameter); SSP: Single stable peak Ornstein-Uhlenbeck model with centralizing tendency towards an optimum (alpha: strength of central attraction); lambda; Pagel’s lambda measuring deviation of inter-tip covariance matrix from expectations of BM (lambda: multiplication factor applied to the off-diagonal covariance matrix elements maximizing similarity to BM); WN: white noise non-phylogenetic model with all data drawn from a common distribution. Also given are log likelihood values of the observed data (LnLik), number of parameters (k) and AICc values, deviation from optimal model (Delta AiCc), and Akaike weights. Models in bold are the favoured models, either by virtue of lowest AIC_c_ scores or are those with fewest parameters within 2 AIC_c_ units of the lowest AICc scores. *denotes parameters estimated at the bounds placed on the optimization procedure i.e. their actual values may be smaller than given

**Fig. 4:**
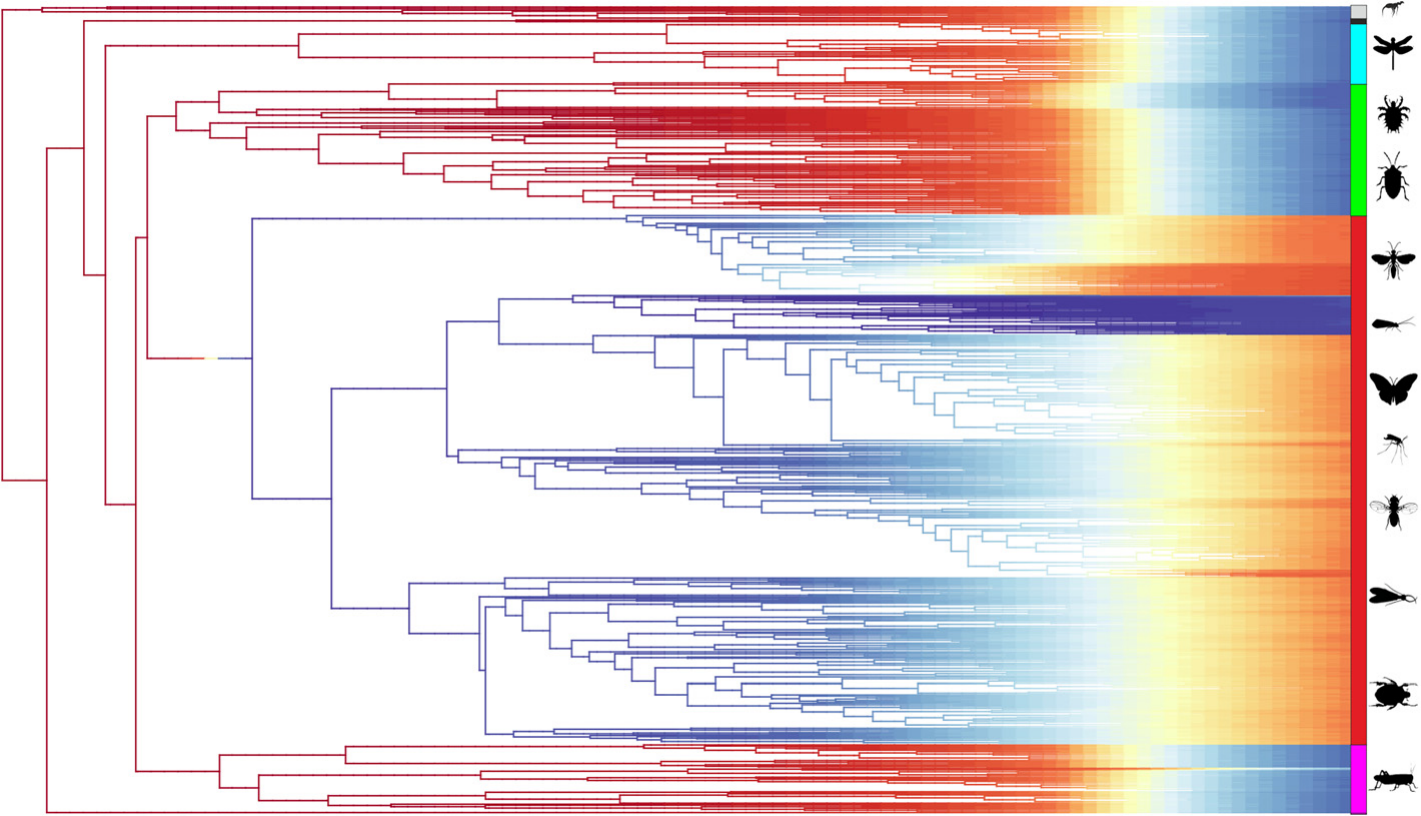
Outputs of Bayesian Analysis of Macroevolutionary Mixtures (BAMM) analysis of log mean body size data. Mean rate of evolution for branches across all post-burnin samples (ln (mm) per million years), denoted by branch coloration (red being high)

The findings of Bayesian Analysis of Macro-evolutionary Mixtures (BAMM) further support the idea that the process of size evolution behaves differently in holometabolan and non-holometabolan groups (Fig. 4). A single shift in the rate model associated with the origins of Holometabola is recovered with a marginal probability of 0.988, i.e. it is found in > 95 % of all sampled models from the post burn-in chain. The single most sampled configuration recovers only this shift (with a relative frequency of 0.5; Additional file 1: Figure S2), suggesting that the impact of other events on size evolution within the group is comparatively marginal. This regime shift in Holometabola is associated with a reversal in the rate of size evolution, such that within this clade rates appear to accelerate through time, contrasting with the weak deceleration observed across the remaining hexapods (potentially consistent with the BM process described above). The only other nodes found to significantly contribute to heterogeneity in size evolution within hexapods are associated with decelerations in size evolution within Trichoptera, both when including (relative frequency 0.17) and when excluding (relative frequency 0.18)) the basal family Hydroptilidae.

## Discussion

The findings of this study corroborate previous taxonomic surveys at continental scales (e.g. [24–26]) suggesting that the distribution of body lengths in hexapod families does not show a strong skew towards an over-abundance of small sized taxa on the log scale. We also demonstrate that, while size does show phylogenetic structuring with respect to different hexapod groups, after accounting for these relationships and the variances observed within tip groups, there is no global negative association between body length and diversification across the studied taxa. Finally, our survey of possible evolutionary models suggests that the pattern and processes of size evolution in Holometabola, and possibly Diptera, are distinct from those of other hexapod groups. In both cases evidence for non-phylogenetic signal suggests that these differences cannot be adequately accounted for in single parameter extensions of Brownian motion, although for other groups, body size evolution looks approximately Brownian.

The recognition that body length distributions in Hexapoda show relatively little bias on a log scale, and that diversification rates within the group are approximately independent of size, supports the idea that concepts derived from the study of vertebrate groups [1, 3] may be inappropriate when discussing other taxonomic groups [5, 6], and hexapods in particular [13, 15, 26]. Possible explanations for these differences focus on the potential for small absolute body size to alter the link between body-size and clade diversification. For example, small-bodied organisms experience distinct flow conditions where viscous forces, such as surface tension and air resistance, have the potential to overwhelm the effect of the gravitational forces (i.e. body weight) that are responsible for structuring body size changes at larger spatial scales [10, 41]. Likewise, fractal environmental models, which postulate the existence of a higher number of niches at small body sizes [8, 9], may become inapplicable below a certain scale, particularly with respect to “parasitic” taxa, which live on the surface of larger host organisms (typical of the majority of hexapods), and are therefore subject to local homogeneity in the composition of their environment across a range of spatial scales [23, 42, 43]. In addition with respect to hexapods, despite a general trend towards larger-bodied organisms showing greater reproductive output, there is evidence from well-studied systems to suggest that this pattern is not universal across the group [17, 44, 45]. Thus, several of the mechanisms typically invoked to account for size-biased diversification in vertebrates may not be applicable to Hexapoda, reflecting a potential danger of extrapolation from well-studied, but atypical clades to describe global evolutionary processes [6]. There is a need to further investigate processes of size evolution across a broader range of invertebrate groups for comparative purposes (e.g. [46]), which, when taken together, may provide us with new insights into underlying mechanisms controlling the size structuring of natural environments [47].

Despite the presence of non-phylogenetic signal in some specific groups, there is considerable evidence that the majority of hexapod clades are strongly phylogenetic structured with respect to body size, and hence size evolution within Hexapoda is broadly described by a BM process on the log scale. However, many specific clades appear, within the limits of available data, to be constrained to a particular subset of possible sizes. The mechanisms underlying such constraint are likely to be variable across different lineages. For example, the absence of small body sizes within Odonata may be attributed to limitations on the minimum size required for the group’s unique flight mechanism [48]. In other cases, the causes of constraint are much less apparent, e.g. the absence of large bodied members of the order Psocodea (booklice; even after accounting for the parasitic and small-bodied Pthiraptera), which may reflect constraints of a cryptic and concealed lifestyle in a group that has received comparatively little detailed study. The effect of such constraints at the super-ordinal scale appears to be marginal, as all of the major lineages demonstrated a wide variation in size as well as homogeneity of process within clades (and across clades, with the exception of Holometabola and Diptera). The overriding impression therefore is that, within the limitations imposed by restricted phylogenetic resolution, size evolution within hexapods is dominated by comparatively localized factors operating at the sub-ordinal or super-familial level.

The reconstruction of estimated standard deviation in body size within Hexapoda generated here bears a strong qualitative resemblance to previously recovered patterns of diversification rate shifts across the clade [30]. This is particularly striking in that clades previously recovered as downshifted with respect to diversification rate, e.g. Psocodea, Neuroptera and Trichoptera, are here recovered as having comparatively low standard deviation in body size, suggesting a link between the diversification process and radiation into novel morphospace [49]. Similar ideas have been previously proposed with respect to bird families, [49], but formalized testing via multiple regression has been shown to be statistically problematic, due to an inability to distinguish time-dependent and speciation-dependent generation of variance [50, 51]. This, in combination with the data abstraction required to treat higher taxonomic groups here (see below; [52]), and the fact that our approaches to estimate standard deviation are confounded with clade richness (see methods; [53]), meant that we did not feel secure in pursuing this line of investigation within the current study. However, in the presence of better data, particularly for within clade body size distributions, this is an intriguing concept and one that merits further investigation.

When considering the processes that may underlie the evolution of hexapod body size, our analyses identify Holometabola and in particular Diptera, as having undergone divergent evolutionary processes when compared with the remaining Hexapoda (the latter being dominated by an overall Brownian drift across the phylogeny). None of the explicit process models explored here were recovered as adequate descriptors of what this divergent process may be, although the BAMM analysis of rate heterogeneity suggests a rate acceleration through time may be involved. The (favored; Table 3) lambda model is not in itself a process description, hence this parameter is most commonly described as a test of phylogenetic signal (e.g. [54]). Despite this limitation, we can conceptually distinguish three possible sources of non-phylogenetic signal that may individually or collectively explain the deviation from BM within these clades: random noise in the dataset (e.g. from inadequate descriptive data), phylogenetic error in taxon assignments, and the presence of complex evolutionary processes that are inadequately accommodated within the single parameter extensions of BM examined above.

Focusing on Diptera as the extreme case of divergence from BM (Additional file 1: Table S3), it can be noted that, in comparison with e.g. Lepidoptera, where the majority of large bodied members are restricted to two derived clades (Macroheterocera; “macro-moths”, and Rhopalocera; butterflies [55]), large bodied flies occur in basal, (e.g. Tipulidae; crane flies), intermediate (e.g. Asilidae and Mydidae; robber and Mydas flies), and highly derived, phylogenetic positions (e.g. Oestridae; bot flies). Likewise, miniaturization also occurs in a range of unrelated families, e.g. Braulidae (bee lice; approximated mean length = 1.30 mm), Corethrellidae (mean =1.22 mm) and Phoridae (mean =1.75 mm), which collectively may further skew size distributions across the order [56]. Thus, there is the potential for divergent processes of size evolution within the clade that are not fully captured by the simplistic evolutionary models implemented here. However, noise in the dataset e.g. from the use of regional taxonomic descriptions (North and Central America [57–59]) as proxies for global size distributions, and phylogenetic uncertainty in relationships, e.g. within Schizophora [30, 60, 61], mean that we should be cautious of over-interpreting these patterns and await better comparative information, preferably incorporating developmental and larval data [13]. It should also be noted that Diptera, and to a lesser extent all Holometabola are, in terms of proportion of probable species described, less well-known than comparable groups (e.g. Coleoptera, Odonata) [62], and thus may be more strongly impacted by collection and modeling biases outlined below.

The apparent association of Holometabola with accelerating rates of size evolution through time (even if we cannot define the specific underlying model) is interesting given that complete metamorphosis has previously been identified as a key innovation in hexapod diversification [30]. Plausible mechanisms for a different process of size evolution within the clade include: modularization of life history stages decoupling adult body-size from larval ecology and so permitting greater adaptive flexibility [13, 31], and historical factors relating to the differential extinction of large bodied non-holometabolan groups [19, 63]. There have been various suggestions, based on the small size of early fossil representatives [33], that patterns within Holometabola may follow the widely acknowledged principal known as Cope’s rule, which postulates that increased niche specialization tends to lead to increased body sizes within a clade over evolutionary time [18] (although in hexapods extreme miniaturization is just as much associated with specialization [14, 22]). However, the lack of a joint systematic framework for extant and fossil taxa has restricted formal testing of this assertion in recent fossil compilations (e.g. [64]).

Unlike well-studied vertebrate clades, there is currently no universal reference source for comparative data within Hexapoda, nor of the demographic or ecological information that may aid in interpreting models of size evolution [36, 65]. As a result, the information used here is derived from a mix of global and regional scale datasets collected at the level of individual clades (Additional file 1: Table S1). This imposes additional assumptions beyond the selection of phylogenetic framework (see discussion of the tree used in [30]) and the use of described species as proxies for total clade richness [66]. There are two major sources of error that may impinge on this analysis and whose extents are problematic to test in the absence of more finely resolved taxonomic data. The first relates to the representative nature of the compiled size limits as accurately reflecting the true size range of studied terminal groups. Due to a lack of data for tropical faunas, the information used here includes an over-reliance on North American, Australian and European taxa, which, due to the presence of a well-known latitudinal cline in insect body size [13], has the potential to bias the raw data on which our findings are based. While acknowledging that such a bias is difficult to explicitly test, we note that previous work has found evidence that regional data for taxonomic groups is predictive of global patterns with respect to hexapod body size [26] and that by combining multiple regional sets we at least attempt to consolidate our size ranges across the known taxonomic range.

A second subtle source of bias originates from the conversion of raw size range data into lognormal distributions that are the source of the parameters used in our modeling procedure. An implicit assumption of using lognormal distributions is that on the logged scale the data is symmetrical around the mean (allowing us to use the observed mean-of-logs as our estimate of average size). However, faunal body size compilations suggest that, with increasing species richness, size distributions becomes increasingly right skewed on the log scale [15], although individual sub-taxa often vary in skew independently of the overall fauna [67]. For the global family distributions considered here, available data on size-distributional skew is insufficiently resolved to contribute to the models considered here, and as a result we have elected to retain the explicit linkage between raw observations and parametric descriptors provided by the assumption of log-normality.

Another difficult-to-test but implicit assumption in our work is that the probability of species description within terminal taxa is not itself biased by body size [68–70] or, to put this another way, that the estimates of described species richness for terminal groups are unbiased approximations of their true extant diversity [66]. The problem of acquiring estimates of “true” species richness based on incomplete records of described species is one of the most profound challenges facing work on any diverse clade (see discussions in [66, 71] and references therein). Of the work conducted here, the observed pattern, i.e. a weak and statistically non-significant positive correlation is potentially consistent with systematic under description of small bodied species; however, this effect would have to be large in-order to mask any “real” negative relationship present within the group. As with many issues relating to unknowns in the richness of large clades, efforts to integrate global taxonomic databases together with associated rates of species description, synonymy resolution and meta-data such as body size, will go a long way towards characterizing what it is that we still do not know regarding hexapod diversity [21].

In addition to description bias, there are also issues relating to the appropriate partitioning of within tip variance, which here we have treated as arising entirely from taxonomic under-sampling. Thus, the effect that novel species description would have on the estimate of the mean body size of a given clade depends on the number of described species in this clade (hence why the estimate of variance is clade-richness dependent [53]), whereas in reality, such estimates also encompass other sources of error such as length variation among individual specimens [72] and sexual dimorphism [73], which may contribute to variation observed across lineages. Dealing with within tip variance in trait measurements is perhaps the greatest outstanding challenge in modeling of trait evolution at deep phylogenetic levels [74]. The methods used here, based on [75, 76], were originally developed with the aim to incorporate measurement error in tip values, with the result that they contain assumptions regarding the distribution of such variance that may not be appropriate for all of the contributing sources of variance present within this dataset. Alternative approaches exist, e.g. “MECCA” [77]; however, these involve simulating multiple species-complete trees (computationally unfeasible on the scale of Hexapoda) and also make strong assumptions regarding variance structure within tip taxa. Further work on partitioning variance within phylogenetic models [74], as well as improved understanding in how such variance is structured in groups where there is good phylogenetic information, represents an area of great potential in understanding how trait evolution may be modeled across very large taxonomic groups.

## Conclusions

Within the limits of the available data and the neontological approach, our analyses suggest that the evolutionary forces structuring macro-evolutionary patterns of body size within Hexapoda are not simply and directly related to those responsible for structuring the diversity of the group. The overall pattern of body size evolution within the group, based on its extant representatives appears to be broadly driven by essentially neutral forces (at a log scale) with the exception of the poorly defined processes operating within Holometabola and Diptera. This conclusion differs from that of fossil based surveys of the group, which have emphasized constraints in shaping size evolution in hexapods, such as oxygen limitation (e.g. [12, 64]) and the evolution of vertebrate predators (notably birds) [78]. These differences reflect differences in the underlying data, including a focus on the evolution of mean body size within clades as opposed to the limits of its maximum value [64], the inability of analyses based on extant data to take account of no-longer existing diversity [79] and impacts of phylogenetic non-independence, which are often neglected in fossil analyses of hexapods [32].

The consequences of these findings for the standard size paradigm (e.g. [1]), with its emphasis on vertebrates, in which size and richness show a strong degree of coupling [2, 3], are significant in that they attack the universality of these findings to other terrestrial clades [6]. As with any macro-evolutionary study involving incompletely described taxonomic groups, we must pay special attention to the role of missing data and interpolation in defining the observed pattern. Hence here we have attempted at a basic level to incorporate within-tip variance into our discussion of body size and diversification. Great challenges remain in trying to tease apart ecological and evolutionary processes in groups operating on temporal and spatial scales profoundly different from our own. The analysis presented here thus should be taken as a step on the road towards a broader understanding of the processes of size evolution and its consequences for an invertebrate perspective of the natural world.

## Methods

An ideal analysis of body size evolution would comprehensively explore patterns and processes at the species level. However, because of the enormous richness of Hexapoda, phylogenetic and trait data are currently too sparse to support a comprehensive species-level analysis. Therefore, for practical reasons we restrict our discussion to the family level, based on recently proposed phylogenetic relationships [30].

All size data for this study is based on family-level estimates of minimum and maximum body length collected from global, regional and taxonomic datasets ([57–59, 80–202], Additional file 1: Table S1). The use of length as a proxy for size is common in Hexapoda due to difficulties in estimating mass from dried museum specimens [13, 15]. Taxon-specific length to mass conversion factors (e.g. [203]) were explored for use in this study and produced qualitatively similar results; however, due to the large amount of uncertainty associated with these values, the presented analyses are restricted to raw length data. Body length was taken as from the anterior margin of the head to the termination of the abdomen, discounting wing cases, abdominal limbs, antennae or cerci where such resolution was available. For taxa such as Lepidoptera (moths) where data-sources record body-size via an alternative metric (e.g. wingspan), average measurements of accompanying illustrations (between one and eight per terminal; selected to encompass the observed diversity) were used to convert these values to body length (examples listed in Additional file 1: Table S1). For Trichoptera (caddis flies), which are typically not illustrated so as to make both the wingspan and body length visible, conversion for the whole order was based on specimens of the various families illustrated in [81].

Estimates of clade richness follow [30]. Resolution of taxonomic conflict is described in Additional file 1: Table S1. In order to avoid issues associated with estimating standard deviation for mono-specific clades (see below) all richness estimates were increased by two for the purposes of modeling relationships. This process is recognized as ad-hoc but regarded as preferable to the loss of phylogenetic information resulting from the exclusion of such lineages. In total, the dataset consisted of 774 terminal taxa spanning all major hexapod lineages (Additional file 1: Table S1).

For modeling purposes, we assumed that, within terminal groups, species conform to a lognormal size-distribution, the parameters of which are estimated from the observed minimum, maximum and richness data. This is a strong assumption, but one conforming to available data regarding hexapod size distributions at the family level [204, 205], and can therefore be regarded as the obvious default in the absence of data to the contrary. The mean of the approximated distributions (henceforth treated on a log scale) was taken as the mean of the log values of the minimum and maximum size estimates (henceforth mean-of-logs). The standard deviation of approximated distributions was estimated using meta-analysis statistics that assume a sample-size dependent relationship between the estimated sd and the observed range [53]. Thus, for very small clades (<15 taxa) sd was calculated using Equation [16] of [53], for moderately diverse groups (16–70 taxa) sd was estimated as range over four, and for large clades (>70 taxa) sd was estimated as range over six [53]. These procedures assume that the mean values for species rich groups are known with greater accuracy (i.e. have smaller associated variance) than species poor groups with the same size-range, reflecting the fact that the former are less likely to be perturbed by further species description (see Discussion). Given that our estimates of standard deviation are thus dependent on corrected clade richness it is appropriate that we maintain this assumption into the derived estimates of standard error (SE) around the clade specific mean-of-logs values. Hence our SE estimates for modeling evolutionary processes [75] were calculated, under the assumption that sample size was equivalent to corrected clade richness.

Descriptive plots of the observed frequency distribution of size were generated for hexapods as a whole and for the major super-ordinal sub-clades [30, 34, 35]. The normality of the overall mean distributions, both at the level of terminal taxa, and with taxa weighted by their observed species richness (Fig. 1), was assessed using an Agostino test [206] (implemented in R [207]; package *moments* [208]). The phylogenetic distribution of minimum, maximum and mean body length, as well as the estimates of terminal standard deviation (Fig. 2, Additional file 1: Figure S1) were plotted using a Brownian motion (BM) ancestral reconstruction [209] implemented in the package *phytools* [210].

The degree of phylogenetic signal present in the data with respect to mean-of-logs size was assessed using Blomberg’s K statistic [211], and by comparing the observed variance among the phylogenetically independent contrasts (PICs) with 1000 randomized data replications, applying the correction of [75] to account for within-group variance (implemented in the package *phytools*) (Table 1). Blomberg’s K can be visualised as measuring the degree to which an observed dataset converges on the expectations of BM (producing an expected value of 1) [211]. Data with no phylogenetic signal will produce a K value of 0 and values less or greater than 1 should be interpreted as lower or higher than expected similarity among terminal taxa, which can be a manifestation of more complex trait evolutionary processes (see below).

To explore the relationship between diversification and body size, we used an adaptation of the PIC derived “macrocaic” method implemented in the package *caper* [212], which is optimized to explore associations of traits values and species richness at the level of higher taxa [38–40]. Richness contrasts at each node were standardized using two metrics: relative rate difference (RRD; Table 2, Fig. 3) and proportion dominance index (PDI; (N_1_/(N_1_ + N_2_)-0.5), Additional file 1: Table S2). Size was modeled as the mean-of-logs estimate and the relationship between the two sets of independent contrasts assessed using regression through the origin [39]. To incorporate within-tip variance in size we used a parametric bootstrap, where across 50,000 pseudo-replicated datasets the values of terminal groups were taken as random draws from the estimated terminal distributions (see above) and the 95 % bounds on the relationship between contrasts were estimated. This distribution was compared with that of an identical number of replicated null data samples where terminal size-values were randomized across the tree. Significance was judged on whether the 95 % confidence intervals on the bootstrapped data excluded those of the randomized null data.

To explore the processes responsible for generating the observed size distribution we used a model testing framework: fitContinuous, in the package *geiger* [213, 214]. Candidate models fitted were: a simple BM process; the early burst model (EB/ACDC), [20, 211] where rates of evolution through time exponentially increase or decrease; the delta model [54], which scales the phylogeny so as to bias the distribution of rates of trait evolution towards either the root or tips; and the SSP model (single stationary peak; modeled as an Ornstein-Uhlenbeck process) [215], which assumes that trait evolution convergences on a single global optimum value (Table 3, Additional file 1: Table S3). All of these models are capable of expressing BM as a special case, resulting from near-zero estimates of the relevant scaling parameters.

In addition, we also fitted two models without an explicit generating process, in order to measure the role of noise and non-phylogenetic signal in the structure of our dataset. The lambda model [54] calculates a global statistic measuring the extent of deviation in the inter-tip covariance matrix from the assumptions of BM (which corresponds to a lambda value of 1). The white noise model (WN) corresponds to a lambda value of 0, and reflects the result that would be obtained in the absence of any phylogenetic structure (star tree) with tip states being drawn from a single underlying normal distribution (Table 3, Additional file 1: Table S3). All fitted models incorporated estimates of standard error around the mean-of-logs, using the methodology of [75] (see above for how these are calculated). Model selection was performed on the basis of AICc values and Akaike weights, see discussion in [20].

Finally, we conducted an exploration of the homogeneity of the process of size evolution within hexapods using the shift-based reversible jump Markov Chain Monte Carlo framework BAMM [216]. As implemented here, the analysis fits EB/ACDC models of size evolution to nodes within the tree signifying regime changes among descendent clades based on an underlying Poisson proposal mechanism. This allows the identification of potential breakpoints in the underlying process of size evolution without the imposition of an explicit prior model. Note that this procedure in its current form is unable to accommodate error in the tip value estimates, thus only the mean-of-log size values for terminal clades were modeled.

Starting values for BAMM were calculated as a homogenous BM process in fitContinuous (betaInit = 0.002424, betaShiftInit = 0), and prior distributions calculated using the package *BAMMtool*s (poissonRatePrior = 1, betaInitPrior = 412.47 betaShiftPrior = 0.002408). We set informative priors on the rate of regime change favoring a homogenous diversification process in order to maximize the credibility of any shifts recovered. Chains were run for 500 million generations with sampling conducted every 5 million generations. Burn-in was estimated based on the stabilization of the inferred likelihood measurements at 10 % of the total sample. Adequate sampling of the stable distribution was assessed on the convergence of two independent runs from divergent starting parameters, based on complete overlap of the credible shift set of models accounting for 70 % of the overall described likelihood. The results presented here are taken only from the first chain, based on the estimated homogenous BM parameters.

## Availability of supporting data

The dataset supporting the results of this article is available in Additional file 1: Table S1.

## Additional file

Additional file 1:
**Figure S1.** Phylogenetic plot of (log) size traits. A) log maximum body length; B) log minimum body length. Ancestral reconstruction of internal nodes based on a BM process (ancML) (Revel [209]). Lower bars denote the minimum and maximum values of observed traits (ln (mm)); coloration on a red to blue scale. Terminal bars denote membership of major clades; colors as in Fig. 1. **Figure S2.** Maximum credible model set from Bayesian Analysis of Macroevolutionary Mixtures (BAMM) corresponding to 95 % of the overall model likelihood. Models are listed in order of frequency (f) of obtaining model in the post burnin set corresponding to their inferred probability (listed from top, left to right). Coloration and tree orientation are as in Fig. 4. **Table S1.** Compiled body length data for included terminal groups with references. Species richness estimates taken from (Rainford et al., [30]); SI. Where multiple references are given they refer respectively to the minimum /maximum values. Taxonomic alterations from (Rainford et al., [30]) are listed in notes. **Table S2.** Outputs of Macrocaic analysis of relationship between PIC of diversification rate (measured as PDI) and mean log size for major clades. **Table S3.** Parameter estimates and relative likelihoods of alternative models of mean body size for major orders of Holometabola (including terminal standard error). Models and parameters denoted as in Table 3. (DOCX 2358 kb)
