# Supplementary material for: Phylogenetic analyses suggest that diversification and body size evolution are independent in insects
Source: BMC Evol Biol. 2016 Jan 8;16:8. doi: 10.1186/s12862-015-0570-3 (PMC4706648; doi:10.1186/s12862-015-0570-3)
Supplement: Additional file 1: — Figure S1. Phylogenetic plot of (log) size traits. A) log maximum body length; B) log minimum body length. Ancestral reconstruction of internal nodes based on a BM process (ancML) (Revel [209]). Lower bars denote the minimum and maximum values of observed traits (ln (mm)); coloration on a red to blue scale. Terminal bars denote membership of major clades; colors as in Fig. 1. Figure S2. Maximum credible model set from Bayesian Analysis of Macroevolutionary Mixtures (BAMM) corresponding to 95 % of the overall model likelihood. Models are listed in order of frequency (f) of obtaining model in the post burnin set corresponding to their inferred probability (listed from top, left to right). Coloration and tree orientation are as in Fig. 4. Table S1. Compiled body length data for included terminal groups with references. Species richness estimates taken from (Rainford et al., [30]); SI. Where multiple references are given they refer respectively to the minimum /maximum values. Taxonomic alterations from (Rainford et al., [30]) are listed in notes. Table S2. Outputs of Macrocaic analysis of relationship between PIC of diversification rate (measured as PDI) and mean log size for major clades. Table S3. Parameter estimates and relative likelihoods of alternative models of mean body size for major orders of Holometabola (including terminal standard error). Models and parameters denoted as in Table 3. (DOCX 2358 kb) [file 12862_2015_570_MOESM1_ESM.docx]

Supplementary Material


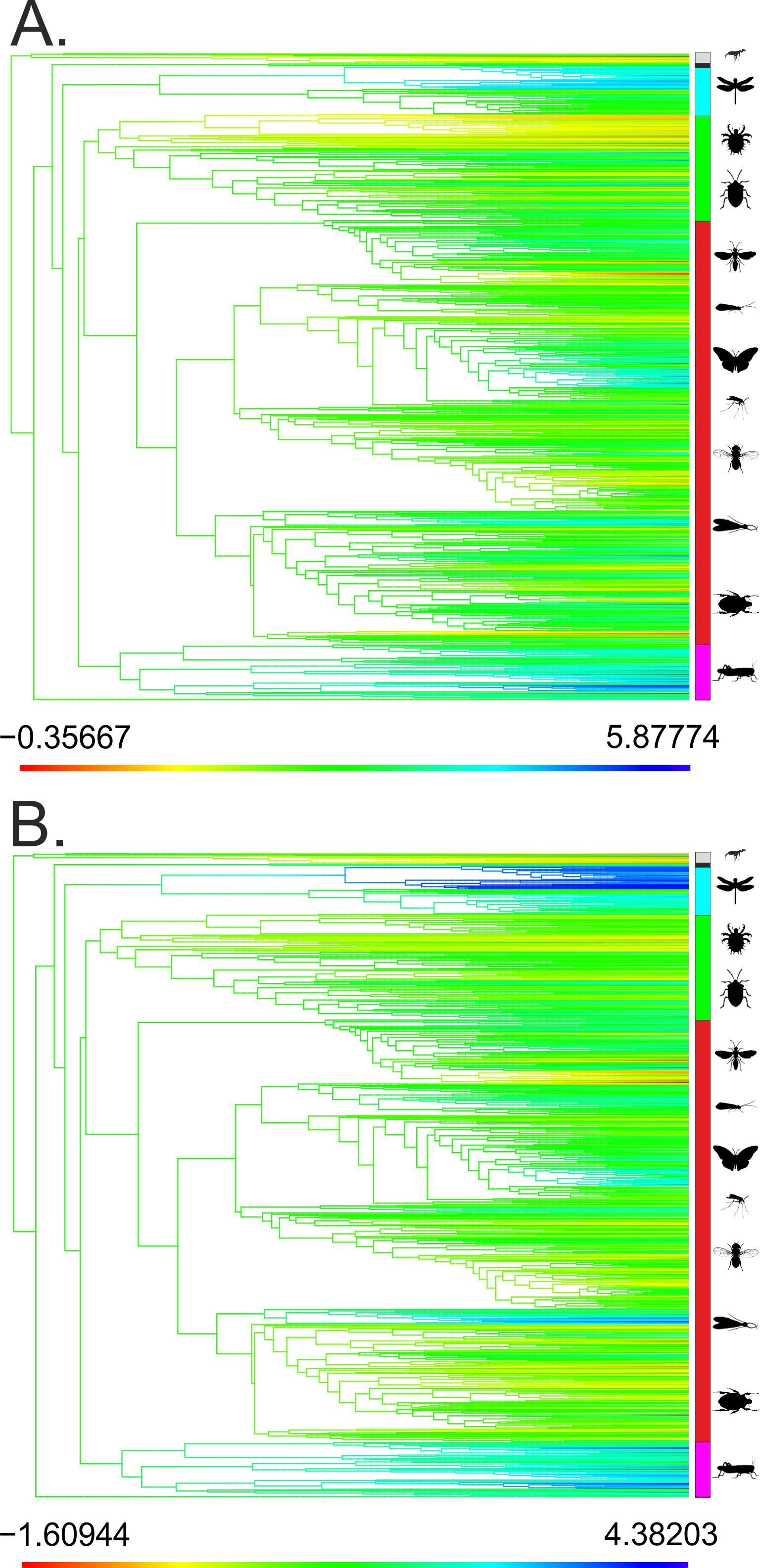


Figure S1. Phylogenetic plot of (log) size traits. A) log maximum body length; B) log minimum body length. Ancestral reconstruction of internal nodes based on a BM process (ancML) (Revel 2013). Lower bars denote the minimum and maximum values of observed traits (ln(mm)); coloration on a red to blue scale. Terminal bars denote membership of major clades; colors as in Figure 1.


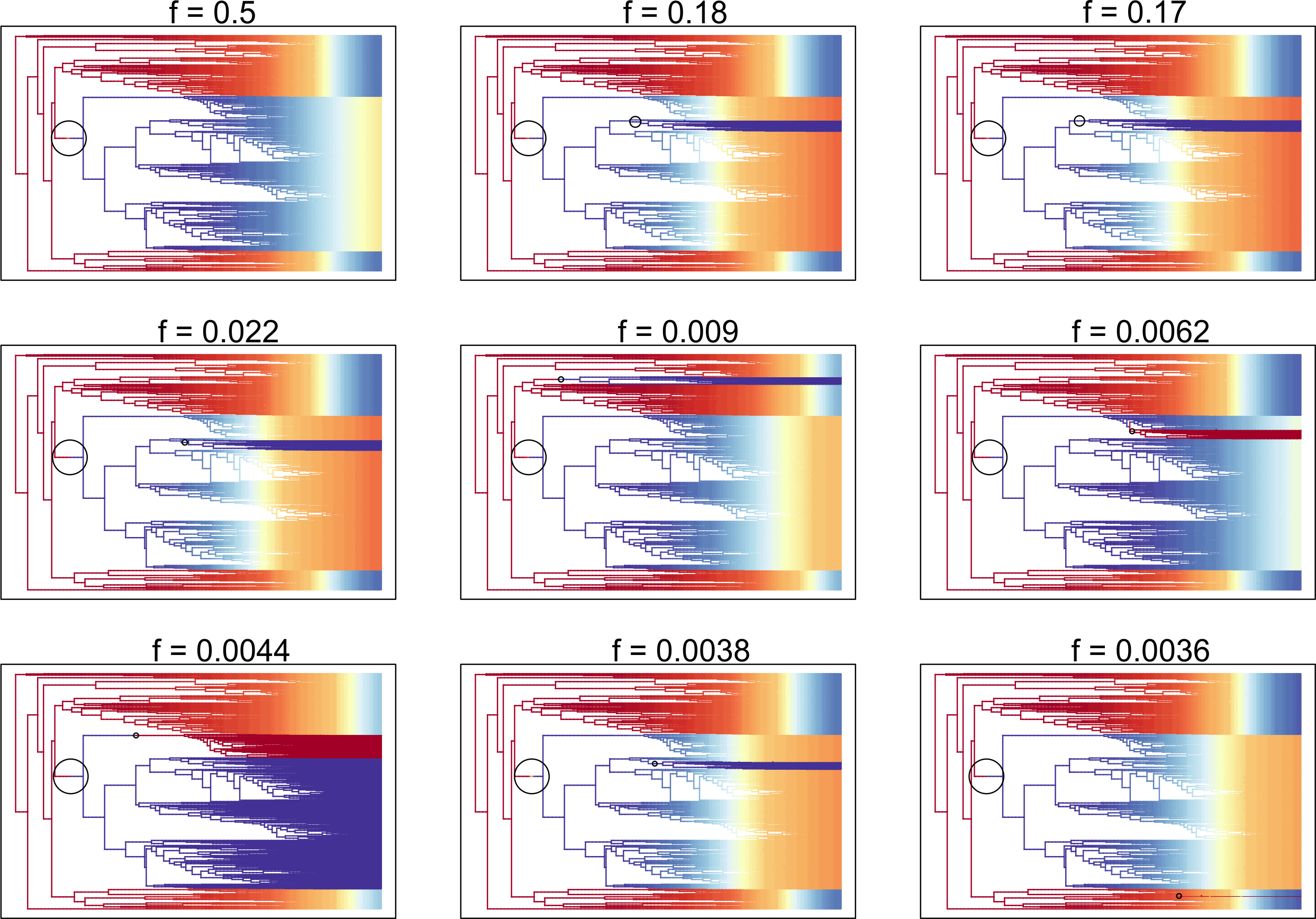


Figure S2. Maximum credible model set from Bayesian Analysis of Macroevolutionary Mixtures (BAMM) corresponding to 95% of the overall model likelihood. Models are listed in order of frequency (f) of obtaining model in the post burnin set corresponding to their inferred probability (listed from top, left to right). Coloration and tree orientation are as in Figure 4.

Table S1. Compiled body length data for included terminal groups with references. Species richness estimates taken from (Rainford *et al.*, 2014); SI. Where multiple references are given they refer respectively to the minimum /maximum values. Taxonomic alterations from (Rainford *et al.*, 2014) are listed in notes.

| Taxon | Richness | Length Data | | Raw Data | | Reference | Notes |
| --- | --- | --- | --- | --- | --- | --- | --- |
|  |  | Min (mm) | Max (mm) | Min (mm) | Max (mm) |  |  |
| Archaeognatha | 495 | 10 | 12 |  |  | (Arnett, 2000) |  |
| Blattodea Blaberidae | 1198 | 2.5 | 75 |  |  | (Arnett, 2000)/(Hogue, 1993) |  |
| Blattodea Blattidae | 2381 | 18 | 45 |  |  | (Arnett, 2000) |  |
| Blattodea Cryptocercidae | 594 | 24 | 29 |  |  | (Arnett, 2000) |  |
| Blattodea Ectobiidae | 12 | 8 | 18 |  |  | (Arnett, 2000) |  |
| Blattodea Corydiidae | 247 | 15 | 24 |  |  | (Arnett, 2000) | Includes Nocticolidae |
| Coleoptera Amphizoidae | 5 | 11 | 16 |  |  | (Parker, 1982) |  |
| Coleoptera Aspidytidae | 2 | 4.8 | 7 |  |  | (Beutel & Leschen, 2005) |  |
| Coleoptera Carabidae | 40000 | 1 | 85 |  |  | (Beutel & Leschen, 2005) |  |
| Coleoptera Dytiscidae | 4015 | 1 | 48 |  |  | (Beutel & Leschen, 2005) |  |
| Coleoptera Gyrinidae | 882 | 3 | 15 |  |  | (Parker, 1982) |  |
| Coleoptera Haliplidae | 218 | 2 | 6 |  |  | (Parker, 1982) |  |
| Coleoptera Hygrobiidae | 5 | 8 | 10 |  |  | (Parker, 1982) |  |
| Coleoptera Noteridae | 250 | 1 | 5.8 |  |  | (Beutel & Leschen, 2005) |  |
| Coleoptera Trachypachidae | 6 | 3.8 | 7 |  |  | (Beutel & Leschen, 2005) |  |
| Coleoptera Cupedidae | 31 | 5 | 22 |  |  | (Beutel & Leschen, 2005) |  |
| Coleoptera Micromalthidae | 1 | 1.5 | 2.5 |  |  | (Parker, 1982) |  |
| Coleoptera Ommatidae | 6 | 6 | 27 |  |  | (Beutel & Leschen, 2005) |  |
| Coleoptera Lepiceridae | 1 | 1.5 | 2 |  |  | (Beutel & Leschen, 2005) |  |
| Coleoptera Hydroscaphidae | 22 | 1 | 2 |  |  | (Beutel & Leschen, 2005) |  |
| Coleoptera Sphaeriusidae | 19 | 0.5 | 1.2 |  |  | (Beutel & Leschen, 2005) |  |
| Coleoptera Torridincolidae | 60 | 1 | 2.7 |  |  | (Beutel & Leschen, 2005) |  |
| Coleoptera Aderidae | 900 | 1 | 4 |  |  | (Arnett *et al.*, 2010) |  |
| Coleoptera Agyrtidae | 70 | 4 | 14 |  |  | (Arnett et al., eds, 2000) |  |
| Coleoptera Alexiidae | 50 | 1.2 | 1.7 |  |  | (Shockley, 2008) |  |
| Coleoptera Anobiidae | 2084 | 1 | 9 |  |  | (Parker, 1982) |  |
| Coleoptera Anthicidae | 3000 | 1.5 | 15 |  |  | (Parker, 1982) |  |
| Coleoptera Anthribidae | 3900 | 1 | 20 |  |  | (Parker, 1982) |  |
| Coleoptera_Polyphaga_ Artematopodidae | 45 | 2.5 | 10 |  |  | (Leschen *et al.*, 2010) |  |
| Coleoptera Attelabidae | 2500 | 1 | 18 |  |  | (Parker, 1982) |  |
| Coleoptera Belidae | 375 | 4.5 | 20 |  |  | (Parker, 1982) |  |
| Coleoptera Biphyllidae | 200 | 1.5 | 8 |  |  | (Parker, 1982) |  |
| Coleoptera Boridae | 4 | 8 | 25 |  |  | (Arnett *et al.*, 2010) |  |
| Coleoptera Bostrichidae | 570 | 1 | 50 |  |  | (Parker, 1982) |  |
| Coleoptera Bothrideridae | 400 | 1.5 | 13 |  |  | (Arnett *et al.*, 2010) |  |
| Coleoptera Brachyceridae | 385 | 1.5 | 6 |  |  | (Arnett *et al.*, 2010) |  |
| Coleoptera Brentidae | 4000 | 3 | 80 |  |  | (Parker, 1982) |  |
| Coleoptera Buprestidae | 14700 | 1.5 | 60 |  |  | (Parker, 1982) |  |
| Coleoptera Byrrhidae | 430 | 1.5 | 10 |  |  | (Parker, 1982) |  |
| Coleoptera Byturidae | 24 | 2.5 | 8 |  |  | (Parker, 1982) |  |
| Coleoptera Callirhipidae | 150 | 9 | 23 |  |  | (Arnett *et al.*, 2010) |  |
| Coleoptera Cantharidae | 5100 | 1.5 | 30 |  |  | (Parker, 1982) |  |
| Coleoptera Cephaloidae | 19 | 4.2 | 22 |  |  | (Leschen *et al.*, 2010) |  |
| Coleoptera Cerambycidae | 30079 | 2 | 200 |  |  | (Parker, 1982) |  |
| Coleoptera Ceratocanthidae | 120 | 2 | 9 |  |  | (Arnett *et al.*, 2010) |  |
| Coleoptera Cerylonidae | 450 | 1 | 4 |  |  | (Parker, 1982) |  |
| Coleoptera Chelonariidae | 250 | 2.5 | 10 |  |  | (Parker, 1982) |  |
| Coleoptera Chrysomelidae | 32500 | 1 | 40 |  |  | (Parker, 1982) |  |
| Coleoptera Ciidae | 650 | 0.5 | 7 |  |  | (Parker, 1982) |  |
| Coleoptera Clambidae | 170 | 0.7 | 2 |  |  | (Parker, 1982) |  |
| Coleoptera Cleridae | 3400 | 2 | 25 |  |  | (Parker, 1982) |  |
| Coleoptera Coccinellidae | 6000 | 1 | 10 |  |  | (Parker, 1982) |  |
| Coleoptera Corylophidae | 200 | 0.7 | 2.3 |  |  | (Parker, 1982) |  |
| Coleoptera Cryptophagidae | 600 | 1 | 4 |  |  | (Parker, 1982) |  |
| Coleoptera Cucujidae | 44 | 2.5 | 25 |  |  | (Leschen *et al.*, 2010) |  |
| Coleoptera Curculionidae | 50615 | 1 | 55 |  |  | (Parker, 1982) |  |
| Coleoptera Dascillidae | 80 | 6 | 20 |  |  | (Parker, 1982) |  |
| Coleoptera Dermestidae | 1200 | 1 | 12 |  |  | (Parker, 1982) |  |
| Coleoptera Derodontidae | 30 | 1.5 | 4 |  |  | (Parker, 1982) |  |
| Coleoptera Discolomatidae | 400 | 1.1 | 8 |  |  | (Parker, 1982) |  |
| Coleoptera Drilidae | 120 | 3 | 10 |  |  | (Parker, 1982) |  |
| Coleoptera Dryopidae | 300 | 2 | 8 |  |  | (Parker, 1982) |  |
| Coleoptera Elateridae | 10000 | 1.5 | 60 |  |  | (Parker, 1982) |  |
| Coleoptera Elmidae | 1500 | 1 | 8 |  |  | (Parker, 1982) |  |
| Coleoptera Endomychidae | 1800 | 1 | 18 |  |  | (Parker, 1982) |  |
| Coleoptera Epimetopidae | 27 | 1 | 4 |  |  | (Beutel & Leschen, 2005) |  |
| Coleoptera Erotylidae | 2500 | 2.5 | 25 |  |  | (Parker, 1982) |  |
| Coleoptera Eucinetidae | 53 | 0.8 | 4 |  |  | (Parker, 1982) |  |
| Coleoptera Eucnemidae | 1500 | 1.5 | 40 |  |  | (Leschen *et al.*, 2010) |  |
| Coleoptera Eulichadidae | 30 | 15 | 25 |  |  | (Parker, 1982) |  |
| Coleoptera Georissidae | 77 | 1 | 3 |  |  | (Parker, 1982) |  |
| Coleoptera Geotrupidae | 920 | 5 | 45 |  |  | (Arnett *et al.*, 2010) |  |
| Coleoptera Glaphyridae | 204 | 6 | 20 |  |  | (Arnett *et al.*, 2010) |  |
| Coleoptera Glaresidae | 57 | 2.5 | 6 |  |  | (Arnett *et al.*, 2010) |  |
| Coleoptera Helophoridae | 183 | 2 | 9 |  |  | (Beutel & Leschen, 2005) |  |
| Coleoptera Helotidae | 107 | 6 | 16 |  |  | (Parker, 1982) |  |
| Coleoptera Heteroceridae | 300 | 1 | 8 |  |  | (Arnett *et al.*, 2010) |  |
| Coleoptera Histeridae | 4300 | 0.5 | 20 |  |  | Parker et al 82 |  |
| Coleoptera Hybosoridae | 572 | 5 | 7 |  |  | (Arnett *et al.*, 2010) |  |
| Coleoptera Hydraenidae | 1600 | 1.2 | 3 |  |  | (Parker, 1982) |  |
| Coleoptera Hydrochidae | 164 | 2 | 4 |  |  | (Jäch & Balke, 2003) |  |
| Coleoptera Hydrophilidae | 3400 | 1 | 40 |  |  | (Parker, 1982) |  |
| Coleoptera Ithyceridae | 6 | 12 | 15 |  |  | (Parker, 1982) |  |
| Coleoptera Kateretidae | 95 | 1.3 | 6 |  |  | (Hisamatsu, 2011) |  |
| Coleoptera Laemophloeidae | 430 | 1 | 5 |  |  | (Arnett *et al.*, 2010) |  |
| Coleoptera Lampyridae | 2200 | 4 | 30 |  |  | (Parker, 1982) |  |
| Coleoptera Languriidae | 1000 | 1.2 | 25 |  |  | (Parker, 1982) |  |
| Coleoptera Latridiidae | 1000 | 1 | 3 |  |  | (Shockley *et al.*, 2011) |  |
| Coleoptera Leiodidae | 3700 | 1 | 7 |  |  | (Parker, 1982) |  |
| Coleoptera Limnichidae | 390 | 0.8 | 3 |  |  | (Parker, 1982) |  |
| Coleoptera Lucanidae | 1489 | 4 | 80 |  |  | (Parker, 1982) |  |
| Coleoptera Lutrochidae | 11 | 3 | 5 |  |  | (Parker, 1982) |  |
| Coleoptera Lycidae | 4600 | 3 | 22 |  |  | (Parker, 1982) |  |
| Coleoptera Lymexylidae | 70 | 5 | 40 |  |  | (Parker, 1982) |  |
| Coleoptera Mauroniscidae | 26 | 2 | 4.5 |  |  | (Leschen *et al.*, 2010) |  |
| Coleoptera Melandryidae | 420 | 1.2 | 19 |  |  | (Parker, 1982) |  |
| Coleoptera Meloidae | 3000 | 5 | 33 |  |  | (Parker, 1982) |  |
| Coleoptera Melyridae | 6000 | 1 | 20 |  |  | (Parker, 1982) |  |
| Coleoptera Monotomidae | 250 | 1.3 | 5 |  |  | (Parker, 1982) |  |
| Coleoptera Mordellidae | 1500 | 2 | 15 |  |  | (Parker, 1982) |  |
| Coleoptera Mycetophagidae | 130 | 0.8 | 6.5 |  |  | (Parker, 1982) |  |
| Coleoptera Nemonychidae | 70 | 4 | 6 |  |  | (Parker, 1982) |  |
| Coleoptera Nitidulidae | 4500 | 0.9 | 14 |  |  | (Parker, 1982) |  |
| Coleoptera Nosodendridae | 50 | 2.5 | 9 |  |  | (Parker, 1982) |  |
| Coleoptera Ochodaeidae | 110 | 3 | 10 |  |  | (Arnett *et al.*, 2010) |  |
| Coleoptera Oedemeridae | 500 | 5 | 20 |  |  | (Parker, 1982) |  |
| Coleoptera Omalisidae | 8 | 3 | 9 |  |  | (Leschen *et al.*, 2010) |  |
| Coleoptera Omethidae | 33 | 3 | 12 |  |  | (Arnett *et al.*, 2010) |  |
| Coleoptera Orsodacnidae | 40 | 4 | 15 |  |  | (Arnett *et al.*, 2010) |  |
| Coleoptera Passalidae | 800 | 18 | 80 |  |  | (Parker, 1982) |  |
| Coleoptera Passandridae | 109 | 3 | 35 |  |  | (Leschen *et al.*, 2010) |  |
| Coleoptera Perimylopidae | 19 | 6 | 10 |  |  | (Parker, 1982) |  |
| Coleoptera Phalacridae | 640 | 1.2 | 4.5 |  |  | (Parker, 1982) |  |
| Coleoptera Phengodidae | 250 | 3 | 65 |  |  | (Parker, 1982) |  |
| Coleoptera Phloeostichidae | 14 | 2.4 | 15 |  |  | (Leschen *et al.*, 2010) |  |
| Coleoptera Phloiophilidae | 1 | 2 | 3 |  |  | (Parker, 1982) |  |
| Coleoptera Pleocomidae | 50 | 15 | 45 |  |  | (Parker, 1982) |  |
| Coleoptera Prionoceridae | 160 | 5.5 | 20 |  |  | (Leschen *et al.*, 2010) |  |
| Coleoptera Propalticidae | 30 | 1.2 | 1.8 |  |  | (Parker, 1982) |  |
| Coleoptera Prostomidae | 30 | 5 | 10 |  |  | (Arnett *et al.*, 2010) |  |
| Coleoptera Protocucujidae | 7 | 3.5 | 5.8 |  |  | (Leschen *et al.*, 2010) |  |
| Coleoptera Psephenidae | 290 | 2 | 7 |  |  | (Parker, 1982) |  |
| Coleoptera Ptiliidae | 650 | 0.3 | 2 |  |  | (Parker, 1982) |  |
| Coleoptera Ptilodactylidae | 500 | 2 | 16 |  |  | (Parker, 1982) |  |
| Coleoptera Ptinidae | 500 | 1 | 5 |  |  | (Parker, 1982) |  |
| Coleoptera Pyrochroidae | 167 | 7 | 18 |  |  | (Parker, 1982) |  |
| Coleoptera Pythidae | 23 | 3 | 20 |  |  | (Parker, 1982) |  |
| Coleoptera Rhipiceridae | 70 | 10 | 25 |  |  | (Parker, 1982) |  |
| Coleoptera Ripiphoridae | 400 | 2 | 38 |  |  | (Parker, 1982) |  |
| Coleoptera Salpingidae | 300 | 1.5 | 12 |  |  | (Parker, 1982) |  |
| Coleoptera Scarabaeidae | 27000 | 1 | 160 |  |  | (Parker, 1982) |  |
| Coleoptera Scirtidae | 800 | 1.5 | 12 |  |  | (Parker, 1982) |  |
| Coleoptera Scraptiidae | 500 | 1.3 | 15 |  |  | (Parker, 1982) |  |
| Coleoptera Scydmaenidae | 4586 | 0.5 | 7 |  |  | (Parker, 1982) |  |
| Coleoptera Silphidae | 200 | 7 | 45 |  |  | (Parker, 1982) |  |
| Coleoptera Silvanidae | 500 | 2 | 15 |  |  | (Arnett *et al.*, 2010) |  |
| Coleoptera Spercheidae | 19 | 3 | 7 |  |  | (Darilmaz & Kiyak, 2011) |  |
| Coleoptera Sphaeritidae | 5 | 4 | 6 |  |  | (Parker, 1982) |  |
| Coleoptera Sphindidae | 59 | 1.5 | 3.5 |  |  | (Leschen *et al.*, 2010) |  |
| Coleoptera Staphylinidae | 56000 | 0.5 | 50 |  |  | (Parker, 1982) |  |
| Coleoptera Synchroidae | 8 | 7 | 13 |  |  | (Parker, 1982) |  |
| Coleoptera Tenebrionidae | 20000 | 1 | 50 |  |  | (Parker, 1982) |  |
| Coleoptera Tetratomidae | 150 | 2.8 | 15 |  |  | (Parker, 1982) |  |
| Coleoptera Throscidae | 150 | 1.2 | 6 |  |  | (Leschen *et al.*, 2010) |  |
| Coleoptera Trictenotomidae | 13 | 32 | 80 |  |  | (Leschen *et al.*, 2010) |  |
| Coleoptera Trogossitidae | 600 | 1 | 50 |  |  | (Parker, 1982) |  |
| Coleoptera Zopheridae | 1700 | 2 | 40 |  |  | (Parker, 1982) |  |
| Collembola Entomobryidae | 2189 | 1 | 10 |  |  | (Arnett, 2000) | Includes Paronellidae- |
| Collembola Hypogastruridae | 682 | 0.8 | 3 |  |  | (Arnett, 2000) |  |
| Collembola Isotomidae | 1346 | 0.7 | 6 |  |  | (Arnett, 2000) |  |
| Collembola Neanuridae | 1546 | 2 | 3.5 |  |  | (Arnett, 2000) | includes Brachystomellidae |
| Collembola Neelidae | 33 | 0.3 | 0.7 |  |  | (Arnett, 2000) |  |
| Collembola Onychiuridae | 913 | 0.5 | 3 |  |  | (Arnett, 2000) | includes Odontellidae + Tullbergiidae |
| Collembola Poduridae | 1 | 1.3 | 2 |  |  | (Arnett, 2000)/(Hopkin, 1997) |  |
| Collembola Sminthuridae | 742 | 0.4 | 2.7 |  |  | (Arnett, 2000) | includes Bourletiellidae, Dicyrtomidae and Oncopoduridae |
| Collembola Tomoceridae | 354 | 6 | 10 |  |  | (Arnett, 2000) | includes Katiannidae |
| Dermaptera Anisolabididae | 38 | 9 | 13 |  |  | (Arnett, 2000) |  |
| Dermaptera Apachyidae | 15 | 11 | 25 |  |  | (Boeseman, 1954) |  |
| Dermaptera Chelisochidae | 95 | 16 | 20 |  |  | (Arnett, 2000) |  |
| Dermaptera Forficulidae | 485 | 10 | 18 |  |  | (Arnett, 2000) |  |
| Dermaptera Labiduridae | 64 | 18 | 80 |  |  | (Arnett, 2000)/(Berenbaum, 2007) |  |
| Dermaptera Labiidae | 495 | 4 | 7 |  |  | (Arnett, 2000) |  |
| Dermaptera Pygidicranidae | 181 | 9 | 45 |  |  | (Parker, 1982) |  |
| Diplura Campodeidae | 448 | 8 | 10 |  |  | (Arnett, 2000) |  |
| Diplura Japygoidea | 590 | 8 | 50 |  |  | (Arnett, 2000) |  |
| Diptera Acartophthalmidae | 6 | 2.5 | 3 |  |  | (McAlpine *et al.*, eds, 1987) |  |
| Diptera Acroceridae | 400 | 2 | 21 |  |  | (Brown *et al.*, 2009) |  |
| Diptera Agromyzidae | 3017 | 0.9 | 6.5 |  |  | (Brown *et al.*, 2009) |  |
| Diptera Anisopodidae | 196 | 2 | 18 |  |  | (McAlpine *et al.*, eds, 1981) |  |
| Diptera Anthomyiidae | 1941 | 2 | 12 |  |  | (McAlpine *et al.*, eds, 1987) |  |
| Diptera Anthomyzidae | 100 | 1.1 | 3.4 |  |  | (Brown *et al.*, 2009) |  |
| Diptera Apioceridae | 143 | 7.5 | 35 |  |  | (McAlpine *et al.*, eds, 1981) |  |
| Diptera Apsilocephalidae | 7 | 4.5 | 5.5 |  |  | (Nagatomi *et al.*, 1991) |  |
| Diptera Asilidae | 7531 | 3 | 60 |  |  | (Brown *et al.*, 2009) |  |
| Diptera Asteiidae | 138 | 1 | 5 |  |  | (Brown *et al.*, 2009) |  |
| Diptera Atelestidae | 22 | 1.5 | 4 |  |  | (Wiegmann, 1989)/(Capinera, 2008) |  |
| Diptera Athericidae | 133 | 7 | 10 |  |  | (Brown *et al.*, 2009) |  |
| Diptera Aulacigastridae | 19 | 1.5 | 4 |  |  | (Brown *et al.*, 2009) |  |
| Diptera Australimyzidae | 9 | 1.3 | 2.6 |  |  | (Brake & Mathis, 2007) |  |
| Diptera Austroleptidae | 8 | 3.1 | 5.3 |  |  | (Nagatomi & Nagatomi, 1987) |  |
| Diptera Axymyiidae | 8 | 4 | 7 | 5 | 8 | (Schneeberg *et al.*, 2013) | Data given as wing length |
| Diptera Bibionidae | 1382 | 2 | 15 |  |  | (Brown *et al.*, 2009) | Includes Pleciidae |
| Diptera Blephariceridae | 331 | 3 | 13 |  |  | (McAlpine *et al.*, eds, 1981) |  |
| Diptera Bombyliidae | 5382 | 4 | 40 |  |  | (Arnett, 2000) |  |
| Diptera_Braulidae | 7 | 1 | 1.7 |  |  | (McAlpine *et al.*, eds, 1987) |  |
| Diptera Calliphoridae | 1525 | 4 | 16 |  |  | (McAlpine *et al.*, eds, 1987) |  |
| Diptera Canthyloscelidae | 14 | 2 | 3.5 |  |  | Manual of Neoarctic diptera |  |
| Diptera Carnidae | 92 | 1 | 3 |  |  | (Brown *et al.*, 2009) |  |
| Diptera Cecidomyiidae | 6296 | 1 | 8 |  |  | (McAlpine *et al.*, eds, 1981) |  |
| Diptera Ceratopogonidae | 5902 | 1 | 6 |  |  | (McAlpine *et al.*, eds, 1981) |  |
| Diptera Chaoboridae | 89 | 1.4 | 10 |  |  | (McAlpine *et al.*, eds, 1981) |  |
| Diptera Chironomidae | 7290 | 1 | 13 |  |  | (Brown *et al.*, 2009) |  |
| Diptera Chloropidae | 2885 | 1 | 7 |  |  | (Karpa, 2001) |  |
| Diptera Chyromyidae | 139 | 0.5 | 4.5 |  |  | (Brown *et al.*, 2009)/ (McAlpine *et al.*, eds, 1987) |  |
| Diptera Clusiidae | 363 | 1.8 | 7.5 |  |  | (McAlpine *et al.*, eds, 1987) |  |
| Diptera Coelopidae | 35 | 3 | 16 |  |  | (Brown *et al.*, 2009) |  |
| Diptera Conopidae | 831 | 2.5 | 30 |  |  | (Brown *et al.*, 2009) |  |
| Diptera Corethrellidae | 111 | 0.6 | 2.5 |  |  | (Brown *et al.*, 2009) |  |
| Diptera Culicidae | 3725 | 3 | 9 |  |  | (McAlpine *et al.*, eds, 1981) |  |
| Diptera Deuterophlebiidae | 14 | 2 | 4 |  |  | (Arnett, 2000) |  |
| Diptera Diadocidiidae | 39 | 3 | 10 |  |  | (Bechev & Chandler, 2011) |  |
| Diptera Diopsidae | 194 | 4 | 12 |  |  | (McAlpine *et al.*, eds, 1987) |  |
| Diptera Dixidae | 197 | 4.5 | 7 |  |  | (Arnett, 2000) |  |
| Diptera Dolichopodidae | 7358 | 0.8 | 9 |  |  | (McAlpine *et al.*, eds, 1981) |  |
| Diptera Drosophilidae | 4017 | 1 | 7 |  |  | (Brown *et al.*, 2009) |  |
| Diptera Dryomyzidae | 30 | 4 | 18 |  |  | (Mathis & Sueyoshi, 2011) |  |
| Diptera Empididae | 3142 | 2 | 12 |  |  | (Capinera, 2008) |  |
| Diptera Ephydridae | 1994 | 0.6 | 11 |  |  | (Brown *et al.*, 2009) |  |
| Diptera Fanniidae | 359 | 3.5 | 7.5 |  |  | (Brown *et al.*, 2009) |  |
| Diptera Fergusoninidae | 29 | 2 | 3 |  |  | (Nelson *et al.*, 2011) |  |
| Diptera Glossinidae | 25 | 6 | 14 |  |  | (Wall & Shearer, 2008) |  |
| Diptera Helcomyzidae | 12 | 3 | 16 |  |  | (Mathis, 2011a) |  |
| Diptera Helosciomyzidae | 23 | 5 | 11 |  |  | (Barnes, 1981) |  |
| Diptera Hesperinidae | 10 | 4.7 | 12 |  |  | (Papp, 2010) |  |
| Diptera Heterocheilidae | 2 | 4.2 | 6.5 |  |  | (Mathis, 2011b) |  |
| Diptera Hilarimorphidae | 36 | 1.8 | 7.2 |  |  | (McAlpine *et al.*, eds, 1981) |  |
| Diptera Hippoboscidae | 271 | 1.5 | 12 |  |  | (McAlpine *et al.*, eds, 1987) |  |
| Diptera Hybotidae | 2005 | 1 | 9 |  |  | (Capinera, 2008) |  |
| Diptera Keroplatidae | 993 | 2.8 | 8.8 |  |  | (Brown *et al.*, 2009) |  |
| Diptera Lauxaniidae | 1900 | 2 | 11 |  |  | (Brown *et al.*, 2009) |  |
| Diptera Lonchaeidae | 504 | 3 | 6 |  |  | (Brown *et al.*, 2009) |  |
| Diptera Lonchopteridae | 65 | 2 | 4 |  |  | (Brown *et al.*, 2009) |  |
| Diptera Lygistorrhinidae | 44 | 3 | 5 |  |  | (Brown *et al.*, 2009) |  |
| Diptera Marginidae | 3 | 1.5 | 2 |  |  | (McAlpine, 1991) |  |
| Diptera Micropezidae | 583 | 5 | 17 |  |  | (Brown *et al.*, 2009) |  |
| Diptera Milichiidae | 288 | 1 | 7 |  |  | (Brown *et al.*, 2009) |  |
| Diptera Muscidae | 5218 | 2 | 20 |  |  | (Brown *et al.*, 2009) |  |
| Diptera Mycetophilidae | 4525 | 2.2 | 13.3 |  |  | (McAlpine *et al.*, eds, 1981) |  |
| Diptera Mydidae | 498 | 9 | 60 |  |  | (McAlpine *et al.*, eds, 1981) |  |
| Diptera Mythicomyiidae | 350 | 0.8 | 3 |  |  | (Brown *et al.*, 2009) |  |
| Diptera Nemestrinidae | 300 | 4 | 16 |  |  | (Brown *et al.*, 2009) |  |
| Diptera Neurochaetidae | 22 | 1.5 | 4.1 |  |  | (McAlpine, 1993) |  |
| Diptera Nycteribiidae | 274 | 1.5 | 5.5 |  |  | (Brown *et al.*, 2009) |  |
| Diptera Odiniidae | 65 | 2.5 | 6 |  |  | (Brown *et al.*, 2009) |  |
| Diptera Oestridae | 176 | 8 | 25 |  |  | (McAlpine *et al.*, eds, 1987) |  |
| Diptera Opomyzidae | 61 | 2 | 4.4 |  |  | (McAlpine *et al.*, eds, 1987) |  |
| Diptera Pachyneuridae | 8 | 5 | 6 |  |  | (Arnett, 2000) |  |
| Diptera Pallopteridae | 71 | 3 | 5 |  |  | (McAlpine *et al.*, eds, 1987) |  |
| Diptera Pelecorhynchidae | 49 | 4 | 18 |  |  | (McAlpine *et al.*, eds, 1981) |  |
| Diptera Periscelididae | 91 | 2.5 | 5 |  |  | (Brown *et al.*, 2009) |  |
| Diptera Perissommatidae | 9 | 1 | 2 |  |  | (Colless, 1969) |  |
| Diptera Phoridae | 4200 | 0.5 | 6 |  |  | (Brown *et al.*, 2009) |  |
| Diptera Piophilidae | 83 | 3 | 8 |  |  | (Brown *et al.*, 2009) |  |
| Diptera Pipunculidae | 1428 | 2 | 11.5 |  |  | (Brown *et al.*, 2009) |  |
| Diptera Platypezidae | 277 | 1.4 | 10 |  |  | (Brown *et al.*, 2009) |  |
| Diptera Platystomatidae | 1164 | 2.5 | 20 |  |  | (Brown *et al.*, 2009) |  |
| Diptera Psilidae | 322 | 3 | 12 |  |  | (Brown *et al.*, 2009) |  |
| Diptera Psychodidae | 3026 | 1 | 5 |  |  | (Brown *et al.*, 2009) |  |
| Diptera Ptychopteridae | 156 | 7 | 14 |  |  | (Brown *et al.*, 2009) |  |
| Diptera Pyrgotidae | 351 | 5 | 30 |  |  | (Brown *et al.*, 2009) |  |
| Diptera Rhagionidae | 756 | 4 | 12 |  |  | (Brown *et al.*, 2009) |  |
| Diptera Rhinophoridae | 174 | 3.5 | 8 |  |  | (Brown *et al.*, 2009) |  |
| Diptera Richardiidae | 178 | 3 | 15 |  |  | (Brown *et al.*, 2009) |  |
| Diptera Sarcophagidae | 3094 | 5 | 25 |  |  | (Brown *et al.*, 2009) |  |
| Diptera Scathophagidae | 419 | 3 | 13 |  |  | (McAlpine *et al.*, eds, 1987) |  |
| Diptera Scatopsidae | 407 | 0.6 | 4.1 |  |  | (McAlpine *et al.*, eds, 1981) |  |
| Diptera Scenopinidae | 420 | 1 | 8.5 |  |  | (Oosterbroek, 1998) |  |
| Diptera Sciaridae | 2455 | 1 | 11 |  |  | (McAlpine *et al.*, eds, 1981) |  |
| Diptera Sciomyzidae | 618 | 2 | 13 |  |  | (Brown *et al.*, 2009) |  |
| Diptera Sepsidae | 345 | 2 | 7 |  |  | (Brown *et al.*, 2009) |  |
| Diptera Simuliidae | 2121 | 1 | 5.5 |  |  | (McAlpine *et al.*, eds, 1981) |  |
| Diptera Somatiidae | 7 | 3.5 | 5 |  |  | (Brown *et al.*, 2009) |  |
| Diptera Sphaeroceridae | 1571 | 0.7 | 6 |  |  | (Brown *et al.*, 2009) |  |
| Diptera Stratiomyidae | 2690 | 2 | 28 |  |  | (Brown *et al.*, 2009) |  |
| Diptera Streblidae | 237 | 0.7 | 5.5 |  |  | (Brown *et al.*, 2009) |  |
| Diptera Strongylophthalmyiidae | 45 | 2 | 6 |  |  | (Palaczyk *et al.*, 2013) |  |
| Diptera Synneuridae | 3 | 2 | 3.5 |  |  | (McAlpine *et al.*, eds, 1981) |  |
| Diptera Syrphidae | 6107 | 4 | 25 |  |  | (Brown *et al.*, 2009) |  |
| Diptera Tabanidae | 4434 | 6 | 30 |  |  | (McAlpine *et al.*, eds, 1981) |  |
| Diptera Tachinidae | 9626 | 3 | 25 |  |  | (Brown *et al.*, 2009) |  |
| Diptera Tanyderidae | 55 | 11 | 23 | 20 | 42 | (Arnett, 2000) | Data given as wingspan |
| Diptera Tephritidae | 4716 | 2 | 35 |  |  | (Brown *et al.*, 2009) |  |
| Diptera Thaumaleidae | 183 | 2 | 4.5 |  |  | (Arnett, 2000) |  |
| Diptera Therevidae | 1143 | 2.5 | 15 |  |  | (McAlpine *et al.*, eds, 1981) |  |
| Diptera Tipulidae | 15770 | 6 | 60 |  |  | (McAlpine *et al.*, eds, 1981) |  |
| Diptera Trichoceridae | 183 | 3 | 9 |  |  | (Brown *et al.*, 2009) |  |
| Diptera Ulidiidae | 678 | 2 | 14 |  |  | (Brown *et al.*, 2009) |  |
| Diptera Vermileonidae | 61 | 7 | 12 |  |  | (Brown *et al.*, 2009) |  |
| Diptera Xenasteiidae | 13 | 1.2 | 2 |  |  | (Evenhius, 2011) |  |
| Diptera Xylomyidae | 138 | 5 | 15 |  |  | (McAlpine *et al.*, eds, 1981) |  |
| Diptera Xylophagidae | 145 | 2 | 25 |  |  | (McAlpine *et al.*, eds, 1981) |  |
| Embioptera | 337 | 4 | 22 |  |  | (Arnett, 2000) | Represents Order |
| Ephemeroptera Ameletidae | 56 | 7 | 21 |  |  | (Zloty & Pritchard, 1997) |  |
| Ephemeroptera Ameletopsidae | 6 | 15.5 | 22 |  |  | (Mercado & Elliot, 2005) |  |
| Ephemeroptera Ametropodidae | 3 | 13 | 15 | 13 | 15 | (Edmunds *et al.*, 1976) | Data as forewing length |
| Ephemeroptera Baetidae | 860 | 3 | 10 |  |  | (Arnett, 2000) |  |
| Ephemeroptera Baetiscidae | 12 | 8 | 16 | 8 | 16 | (Edmunds *et al.*, 1976) | Data as forewing length |
| Ephemeroptera Behningiidae | 7 | 12 | 18 |  |  | (Parker, 1982) |  |
| Ephemeroptera Caenidae | 211 | 2 | 6 |  |  | (Arnett, 2000) |  |
| Ephemeroptera Coloburiscidae | 6 | 13 | 18 |  |  | (Marsh, 2004) |  |
| Ephemeroptera Dipteromimidae | 2 | 13 | 23.5 |  |  | (Tojo & Matsukawa, 2003) |  |
| Ephemeroptera Ephemerellidae | 91 | 5 | 12 |  |  | (Arnett, 2000) |  |
| Ephemeroptera Ephemeridae | 160 | 10 | 32 |  |  | (Parker, 1982) |  |
| Ephemeroptera Euthyplociidae | 19 | 11 | 16 |  |  | (Gillies, 1980) |  |
| Ephemeroptera Heptageniidae | 529 | 4 | 14 |  |  | (Arnett, 2000) |  |
| Ephemeroptera Ichthybotidae | 2 | 19 | 22 |  |  | (Phillips, 1930) |  |
| Ephemeroptera Isonychiidae | 30 | 9 | 16 |  |  | (Arnett, 2000) |  |
| Ephemeroptera Leptohyphidae | 157 | 2 | 10 |  |  | (Dominguez *et al.*, 2006) |  |
| Ephemeroptera Leptophlebiidae | 623 | 4 | 12 | 4 | 14 | (Edmunds *et al.*, 1976) | Data as forewing length |
| Ephemeroptera Metretopodidae | 13 | 9 | 16 | 9 | 16 | (Edmunds *et al.*, 1976) | Data as forewing length |
| Ephemeroptera_Neoephemeridae | 7 | 6 | 13 |  |  | (Bae & McCafferty, 1998) |  |
| Ephemeroptera Nesameletidae | 11 | 10.5 | 16.5 |  |  | (Hitchings & Staniczek, 2003) |  |
| Ephemeroptera Oligoneuriidae | 54 | 6 | 10 | 6 | 10 | (Edmunds *et al.*, 1976) | Data as forewing length |
| Ephemeroptera Oniscigastridae | 8 | 10 | 11 |  |  | (Heckman, 2002) |  |
| Ephemeroptera Palingeniidae | 32 | 15 | 35 |  |  | (Parker, 1982) |  |
| Ephemeroptera Polymitarcyidae | 84 | 12 | 35 |  |  | (Parker, 1982) |  |
| Ephemeroptera Potamanthidae | 23 | 8 | 25 |  |  | (Parker 1982) |  |
| Ephemeroptera Prosopistomatidae | 19 | 1.5 | 4.5 |  |  | (Pearson & Penridge 1979) |  |
| Ephemeroptera Rallidentidae | 1 | 10.5 | 12 |  |  | (Penniket 1966) |  |
| Ephemeroptera Siphlaenigmatidae | 1 | 8 | 9 |  |  | (Penniket 1962) |  |
| Ephemeroptera Siphlonuridae | 49 | 9 | 13 |  |  | (Arnett 2000) |  |
| Ephemeroptera Tricorythidae | 34 | 4 | 6.5 |  |  | (Edmunds et al. 1976) | Data as forewing length |
| Grylloblattidae | 27 | 10 | 30 |  |  | (Arnett 2000) |  |
| Hemiptera Acanthosomatidae | 200 | 6 | 18 |  |  | (Schuh & Slater 1995) |  |
| Hemiptera Achilidae | 503 | 3 | 13 |  |  | (Capinera 2008) |  |
| Hemiptera Achilixiidae | 24 | 4 | 8 |  |  | (Capinera 2008) |  |
| Hemiptera Aetalionidae | 42 | 3 | 30 |  |  | (Deitz et al. 2010) |  |
| Hemiptera Aleyrodoidea | 1560 | 1 | 4 |  |  | (Capinera 2008) |  |
| Hemiptera Alydidae | 250 | 8 | 20 |  |  | (Schuh & Slater 1995) |  |
| Hemiptera Anthocoridae | 600 | 1.4 | 4.5 |  |  | (Schuh & Slater 1995) |  |
| Hemiptera Aphelocheiridae | 400 | 3.5 | 11.5 |  |  | (Schuh & Slater 1995) |  |
| Hemiptera Aphidoidea | 4375 | 1 | 8 |  |  | (Capinera 2008) | Includes Phylloxeroidea |
| Hemiptera Aradidae | 2000 | 3 | 11 |  |  | (Schuh & Slater 1995) |  |
| Hemiptera Belostomatidae | 150 | 9 | 110 |  |  | (Schuh & Slater 1995) |  |
| Hemiptera Berytidae | 100 | 2.5 | 11 |  |  | (Schuh & Slater 1995) |  |
| Hemiptera Caliscelidae | 202 | 1 | 5 |  |  | (Capinera 2008) |  |
| Hemiptera Canopidae | 8 | 5 | 7 |  |  | (Schuh & Slater 1995) |  |
| Hemiptera Cercopidae | 2410 | 5 | 20 |  |  | (Arnett 2000) | Includes Aphrophoridae, Clastopteridae, Machaerotidae |
| Hemiptera Cicadellidae | 20000 | 1.7 | 28 |  |  | (Evans 1966) |  |
| Hemiptera Cicadidae | 1300 | 10 | 100 |  |  | (Capinera 2008) |  |
| Hemiptera Cimicidae | 100 | 2 | 12 |  |  | (Schuh & Slater 1995) |  |
| Hemiptera Cixiidae | 2223 | 3 | 13 |  |  | (Capinera 2008) |  |
| Hemiptera Coccoidea | 8000 | 0.6 | 35 |  |  | (Arnett 2000) |  |
| Hemiptera Colobathristidae | 90 | 6 | 20 |  |  | (Schuh & Slater 1995) |  |
| Hemiptera Coreidae | 1900 | 7 | 45 |  |  | (Schuh & Slater 1995) |  |
| Hemiptera Corixidae | 600 | 2.5 | 15 |  |  | (Schuh & Slater 1995) |  |
| Hemiptera Cydnidae | 617 | 2 | 20 |  |  | (Schuh & Slater 1995) |  |
| Hemiptera Delphacidae | 2029 | 2 | 10 |  |  | (Capinera 2008) |  |
| Hemiptera Derbidae | 1700 | 4 | 11 |  |  | (Capinera 2008)/ (Arnett 2000) |  |
| Hemiptera Dictyopharidae | 731 | 3 | 33 |  |  | (Capinera 2008) |  |
| Hemiptera Dinidoridae | 90 | 9 | 27 |  |  | (Schuh & Slater 1995) |  |
| Hemiptera Dipsocoridae | 30 | 0.8 | 3 |  |  | (Schuh & Slater 1995) |  |
| Hemiptera Enicocephalidae | 400 | 2 | 15 |  |  | (Schuh & Slater 1995) |  |
| Hemiptera Eurybrachyidae | 189 | 7 | 29 |  |  | (Capinera 2008) |  |
| Hemiptera Flatidae | 1446 | 4 | 32 |  |  | (Capinera 2008) |  |
| Hemiptera Fulgoridae | 687 | 4 | 100 |  |  | (Capinera 2008) |  |
| Hemiptera Gelastocoridae | 100 | 7 | 15 |  |  | (Schuh & Slater 1995) |  |
| Hemiptera Gerridae | 620 | 1.6 | 36 |  |  | (Schuh & Slater 1995) |  |
| Hemiptera Hebridae | 150 | 1.3 | 3.7 |  |  | (Schuh & Slater 1995) |  |
| Hemiptera Hermatobatidae | 8 | 2.5 | 4 |  |  | (Schuh & Slater 1995) |  |
| Hemiptera Hydrometridae | 110 | 2.7 | 22 |  |  | (Schuh & Slater 1995) |  |
| Hemiptera Hyocephalidae | 3 | 8 | 15 |  |  | Resh and Carde 2009 |  |
| Hemiptera Idiostolidae | 4 | 5 | 7 |  |  | (Schuh & Slater 1995) |  |
| Hemiptera Issidae | 924 | 2 | 19 |  |  | (Capinera 2008) |  |
| Hemiptera Joppeicidae | 1 | 2.5 | 3 |  |  | (Schuh & Slater 1995) | Modified to avoid zero variance |
| Hemiptera Largidae | 120 | 7 | 55 |  |  | (Arnett 2000)/ (Schuh & Slater 1995) |  |
| Hemiptera Leptopodidae | 40 | 1.8 | 7 |  |  | (Schuh & Slater 1995) |  |
| Hemiptera Lestoniidae | 2 | 3.5 | 5.6 |  |  | (Schuh & Slater 1995) |  |
| Hemiptera Lophopidae | 138 | 5 | 15 |  |  | (Capinera 2008) |  |
| Hemiptera Lyctocoridae | 27 | 2 | 6 |  |  | (Schuh & Slater 1995) |  |
| Hemiptera Lygaeidae | 4400 | 1.2 | 12 |  |  | (Schuh & Slater 1995) |  |
| Hemiptera Macroveliidae | 3 | 2.5 | 5.6 |  |  | (Schuh & Slater 1995) |  |
| Hemiptera Malcidae | 20 | 3 | 4 |  |  | (Schuh & Slater 1995) |  |
| Hemiptera Meenoplidae | 158 | 3 | 7 |  |  | (Capinera 2008) |  |
| Hemiptera Membracidae | 3450 | 2 | 24 |  |  | (Deitz et al. 2010) |  |
| Hemiptera Mesoveliidae | 35 | 1.2 | 4.2 |  |  | (Schuh & Slater 1995) |  |
| Hemiptera Microphysidae | 30 | 1.5 | 3 |  |  | (Schuh & Slater 1995) |  |
| Hemiptera Miridae | 10000 | 2 | 15 |  |  | (Schuh & Slater 1995) |  |
| Hemiptera Nabidae | 400 | 7 | 11 |  |  | (Arnett 2000) |  |
| Hemiptera Naucoridae | 500 | 5 | 20 |  |  | (Schuh & Slater 1995) |  |
| Hemiptera Nepidae | 225 | 15 | 45 |  |  | (Schuh & Slater 1995) |  |
| Hemiptera Nogodinidae | 286 | 4 | 17 |  |  | (Capinera 2008) |  |
| Hemiptera Notonectidae | 350 | 5 | 15 |  |  | (Schuh & Slater 1995) |  |
| Hemiptera Ochteridae | 50 | 4.5 | 9 |  |  | (Schuh & Slater 1995) |  |
| Hemiptera Paraphrynoveliidae | 2 | 1.7 | 2.4 |  |  | (Schuh & Slater 1995) |  |
| Hemiptera Peloridiidae | 12 | 2 | 5 |  |  | (Resh & Cardé 2009) |  |
| Hemiptera Pentatomidae | 4500 | 4 | 20 |  |  | (Schuh & Slater 1995) |  |
| Hemiptera Phloeidae | 3 | 20 | 30 |  |  | (Schuh & Slater 1995) |  |
| Hemiptera Piesmatidae | 40 | 2.5 | 5 |  |  | (Schuh & Slater 1995) |  |
| Hemiptera Plataspidae | 500 | 2 | 20 |  |  | (Schuh & Slater 1995) |  |
| Hemiptera Pleidae | 40 | 1.5 | 3 |  |  | (Schuh & Slater 1995) |  |
| Hemiptera Plokiophilidae | 6 | 1.2 | 3 |  |  | (Schuh & Slater 1995) |  |
| Hemiptera Psylloidea | 2500 | 1 | 8 |  |  | (Capinera 2008) |  |
| Hemiptera Pyrrhocoridae | 225 | 8 | 30 |  |  | (Schuh & Slater 1995) |  |
| Hemiptera Reduviidae | 6700 | 7 | 40 |  |  | (Schuh & Slater 1995) | Includes Phymatidae |
| Hemiptera Rhopalidae | 200 | 4 | 15 |  |  | (Schuh & Slater 1995) |  |
| Hemiptera Ricaniidae | 417 | 4 | 12 |  |  | (Capinera 2008) |  |
| Hemiptera Saldidae | 265 | 2.3 | 7.4 |  |  | (Schuh & Slater 1995) |  |
| Hemiptera Schizopteridae | 120 | 0.8 | 2 |  |  | (Schuh & Slater 1995) |  |
| Hemiptera Scutelleridae | 500 | 5 | 20 |  |  | (Schuh & Slater 1995) |  |
| Hemiptera Stenocephalidae | 30 | 8 | 15 |  |  | (Schuh & Slater 1995) |  |
| Hemiptera Termitaphididae | 9 | 2 | 3 |  |  | (Schuh & Slater 1995) |  |
| Hemiptera Tessaratomidae | 250 | 15 | 40 |  |  | (Foottit & Adler 2009) |  |
| Hemiptera Tettigometridae | 73 | 3 | 11 |  |  | (Capinera 2008) |  |
| Hemiptera Thaumastocoridae | 19 | 2 | 4.6 |  |  | (Schuh & Slater 1995) |  |
| Hemiptera Tingidae | 2000 | 2 | 8 |  |  | (Schuh & Slater 1995) |  |
| Hemiptera Tropiduchidae | 575 | 5 | 13 |  |  | (Capinera 2008) |  |
| Hemiptera Veliidae | 720 | 1 | 10 |  |  | (Schuh & Slater 1995) |  |
| Hemiptera Velocipedidae | 31 | 10 | 15 |  |  | (Schuh & Slater 1995) |  |
| Hymenoptera Agaonidae | 757 | 1 | 3 |  |  | (Parker 1982) |  |
| Hymenoptera Ampulicidae | 200 | 5 | 15 |  |  | (Arnett 2000) |  |
| Hymenoptera Anaxyelidae | 1 | 7.5 | 8 |  |  | (Parker 1982) | Modified to avoid zero variance |
| Hymenoptera Andrenidae | 2938 | 4 | 22 |  |  | (Arnett 2000) |  |
| Hymenoptera Aphelinidae | 1168 | 0.35 | 2.5 |  |  | (Parker 1982) |  |
| Hymenoptera Apidae | 5751 | 3.5 | 27 |  |  | (Arnett 2000) |  |
| Hymenoptera Argidae | 800 | 4 | 15 |  |  | (Parker 1982) |  |
| Hymenoptera Aulacidae | 200 | 1 | 20 |  |  | (Parker 1982) |  |
| Hymenoptera Bethylidae | 2000 | 1 | 20 |  |  | (Parker 1982) |  |
| Hymenoptera Blasticotomidae | 10 | 6 | 10 |  |  | (Parker 1982) |  |
| Hymenoptera Braconidae | 20000 | 2 | 15 |  |  | (Parker 1982) |  |
| Hymenoptera Bradynobaenidae | 200 | 3 | 20 |  |  | (Parker 1982) |  |
| Hymenoptera Cephidae | 80 | 5 | 25 |  |  | (Parker 1982) |  |
| Hymenoptera Ceraphronidae | 350 | 0.5 | 5 |  |  | (Parker 1982) |  |
| Hymenoptera Chalcididae | 1464 | 2 | 12 |  |  | (Parker 1982) |  |
| Hymenoptera Chrysididae | 3000 | 2.5 | 20 |  |  | (Parker 1982) |  |
| Hymenoptera Cimbicidae | 130 | 18 | 25 |  |  | (Arnett 2000) |  |
| Hymenoptera Colletidae | 2545 | 3.5 | 20 |  |  | (Arnett 2000) |  |
| Hymenoptera Crabronidae | 8774 | 6 | 20 |  |  | (Arnett 2000) |  |
| Hymenoptera Cynipidae | 1000 | 1 | 8 |  |  | (Parker 1982) |  |
| Hymenoptera Diapriidae | 2300 | 3 | 15 |  |  | (Parker 1982) |  |
| Hymenoptera Diprionidae | 90 | 5 | 12 |  |  | (Parker 1982) |  |
| Hymenoptera Encyrtidae | 3735 | 0.5 | 5 |  |  | (Parker 1982) |  |
| Hymenoptera Eucharitidae | 423 | 3 | 10 |  |  | (Parker 1982) |  |
| Hymenoptera Eulophidae | 4472 | 1 | 5 |  |  | (Parker 1982) |  |
| Hymenoptera Eupelmidae | 907 | 1 | 8 |  |  | (Parker 1982) |  |
| Hymenoptera Eurytomidae | 1424 | 3 | 5 |  |  | (Parker 1982) |  |
| Hymenoptera Evaniidae | 500 | 2 | 15 |  |  | (Parker 1982) |  |
| Hymenoptera Figitidae | 1500 | 1.5 | 5 |  |  | (Parker 1982) |  |
| Hymenoptera Formicidae | 10000 | 1 | 33 |  |  | (Arnett 2000)/(Lenhart et al. 2013) | Sizes given based on workers |
| Hymenoptera Gasteruptiidae | 420 | 13 | 40 |  |  | (Arnett 2000) |  |
| Hymenoptera Halictidae | 4338 | 4 | 10 |  |  | (Arnett 2000) |  |
| Hymenoptera Heloridae | 7 | 4 | 7 |  |  | (Parker 1982) |  |
| Hymenoptera Ibaliidae | 50 | 8 | 25 |  |  | (Parker 1982) |  |
| Hymenoptera Ichneumonidae | 22000 | 3 | 40 |  |  | (Parker 1982) |  |
| Hymenoptera Liopteridae | 50 | 4 | 15 |  |  | (Parker 1982) |  |
| Hymenoptera Maamingidae | 2 | 1 | 2 |  |  | (Early et al. 2001) |  |
| Hymenoptera Megachilidae | 4120 | 7 | 39 |  |  | (Arnett 2000)/(Messer 1984) |  |
| Hymenoptera Megalodontesidae | 40 | 5 | 20 |  |  | (Parker 1982) |  |
| Hymenoptera Megalyridae | 50 | 4 | 20 |  |  | (Parker 1982) |  |
| Hymenoptera Megaspilidae | 450 | 1 | 5 |  |  | (Parker 1982) |  |
| Hymenoptera Melittidae | 191 | 7 | 12 |  |  | (Arnett 2000) |  |
| Hymenoptera Monomachidae | 20 | 7 | 22 |  |  | (Parker 1982) |  |
| Hymenoptera Mutillidae | 5000 | 3 | 30 |  |  | (Parker 1982) |  |
| Hymenoptera Mymaridae | 1424 | 0.2 | 2 |  |  | (Parker 1982) |  |
| Hymenoptera Mymarommatidae | 9 | 0.3 | 0.8 |  |  | (Gibson et al. 2007) |  |
| Hymenoptera Orussidae | 75 | 5 | 20 |  |  | (Parker 1982) |  |
| Hymenoptera Pamphiliidae | 250 | 8 | 15 |  |  | (Arnett 2000) |  |
| Hymenoptera Pelecinidae | 3 | 30 | 60 |  |  | (Parker 1982) |  |
| Hymenoptera Pergidae | 500 | 7 | 10 |  |  | (Arnett 2000) |  |
| Hymenoptera Perilampidae | 277 | 1.5 | 7 |  |  | (Parker 1982) |  |
| Hymenoptera Platygastridae | 1100 | 0.5 | 5 |  |  | (Parker 1982) |  |
| Hymenoptera Plumariidae | 20 | 3 | 10 |  |  | (Parker 1982) |  |
| Hymenoptera Pompilidae | 4000 | 3 | 60 |  |  | (Parker 1982) |  |
| Hymenoptera Proctotrupidae | 310 | 6 | 8 |  |  | (Parker 1982) |  |
| Hymenoptera Pteromalidae | 3506 | 1 | 4 |  |  | (Parker 1982) |  |
| Hymenoptera Roproniidae | 18 | 8 | 10 |  |  | (Parker 1982) |  |
| Hymenoptera Rotoitidae | 2 | 0.7 | 0.9 |  |  | (Bouček & Noyes 1987) |  |
| Hymenoptera Sapygidae | 80 | 6 | 22 |  |  | (Parker 1982) |  |
| Hymenoptera Scelionidae | 3000 | 0.5 | 15 |  |  | (Parker 1982) |  |
| Hymenoptera Scolebythidae | 3 | 7 | 10 |  |  | (Cambra & Oliveira 2003) |  |
| Hymenoptera Scoliidae | 300 | 8 | 60 |  |  | (Parker 1982) |  |
| Hymenoptera Siricidae | 95 | 20 | 40 |  |  | (Parker 1982) |  |
| Hymenoptera Sierolomorphidae | 10 | 3.5 | 6 |  |  | (Parker 1982) |  |
| Hymenoptera Sphecidae | 724 | 18 | 55 |  |  | (Arnett 2000) |  |
| Hymenoptera Stenotritidae | 21 | 14 | 20.5 |  |  | (Houston 1983) |  |
| Hymenoptera Stephanidae | 200 | 4 | 40 |  |  | (Parker 1982) |  |
| Hymenoptera Tenthredinidae | 4000 | 3 | 20 |  |  | (Parker 1982) |  |
| Hymenoptera Tetracampidae | 50 | 0.5 | 2 |  |  | (Doganler 2003) |  |
| Hymenoptera Tiphiidae | 1500 | 4 | 30 |  |  | (Parker 1982) |  |
| Hymenoptera Torymidae | 986 | 1 | 15 |  |  | (Parker 1982) |  |
| Hymenoptera Trichogrammatidae | 839 | 0.5 | 1 |  |  | (Parker 1982) |  |
| Hymenoptera Trigonalidae | 100 | 8 | 17 |  |  | (Parker 1982) |  |
| Hymenoptera Vanhorniidae | 5 | 3 | 10 |  |  | (Arnett 2000) |  |
| Hymenoptera Vespidae | 4000 | 8 | 25 |  |  | (Arnett 2000) |  |
| Hymenoptera Xiphydriidae | 100 | 7 | 25 |  |  | (Parker 1982) |  |
| Hymenoptera Xyelidae | 50 | 5 | 15 |  |  | (Parker 1982) |  |
| Isoptera | 2658 | 4 | 20 |  |  | (Robinson 2005) | Sizes given based on winged forms |
| Lepidoptera Acanthopteroctetidae | 5 | 4 | 6 | 11 | 16 | (Capinera 2008) | Data given as wingspan |
| Lepidoptera Acrolophidae | 300 | 4 | 28 | 9 | 60 | (Capinera 2008) | Data given as wingspan |
| Lepidoptera Adelidae | 294 | 2 | 11 | 4 | 28 | (Arnett 2000) | Data given as wingspan |
| Lepidoptera Agathiphagidae | 2 | 3 | 5 | 9 | 14 | (Capinera 2008) | Data given as wingspan |
| Lepidoptera Agonoxenidae | 4 | 2 | 6 | 6 | 15 | (Capinera 2008) | Data given as wingspan |
| Lepidoptera Aididae | 6 | 4 | 39 | 10 | 90 | (Capinera 2008) | Data given as wingspan |
| Lepidoptera Alucitidae | 216 | 2 | 10 | 7 | 28 | (Capinera 2008) | Data given as wingspan |
| Lepidoptera Amphisbatidae | 21 | 7 | 8 | 17 | 19 | (Capinera 2008) | Data given as wingspan |
| Lepidoptera Andesianidae | 3 | 10 | 23 | 27 | 61 | (Capinera 2008) | Data given as wingspan |
| Lepidoptera Anomoeotidae | 40 | 5 | 8 | 22 | 31 | (Capinera 2008) | Data given as wingspan |
| Lepidoptera Anthelidae | 94 | 9 | 68 | 22 | 166 | (Capinera 2008) | Data given as wingspan |
| Lepidoptera Apatelodidae | 145 | 10 | 37 | 20 | 74 | (Capinera 2008) | Data given as wingspan |
| Lepidoptera Arctiidae | 6000 | 3 | 44 | 8 | 115 | (Capinera 2008) | Data given as wingspan |
| Lepidoptera Argyresthiidae | 157 | 2 | 5 | 6 | 15 | (Capinera 2008) | Data given as wingspan |
| Lepidoptera Arrhenophanidae | 26 | 5 | 29 | 12 | 69 | (Capinera 2008) | Data given as wingspan |
| Lepidoptera Autostichidae | 585 | 4 | 7 | 10 | 20 | (Capinera 2008) | Data given as wingspan |
| Lepidoptera Batrachedridae | 99 | 2 | 9 | 7 | 28 | (Capinera 2008) | Data given as wingspan |
| Lepidoptera Blastobasidae | 377 | 1 | 11 | 5 | 35 | (Capinera 2008) | Data given as wingspan |
| Lepidoptera Bombycidae | 185 | 10 | 33 | 19 | 64 | (Capinera 2008) | Data given as wingspan |
| Lepidoptera Brachodidae | 137 | 3 | 17 | 8 | 42 | (Capinera 2008) | Data given as wingspan |
| Lepidoptera Brahmaeidae | 44 | 18 | 66 | 50 | 180 | (Capinera 2008) | Data given as wingspan |
| Lepidoptera Bucculatricidae | 297 | 2 | 5 | 5 | 16 | (Capinera 2008) | Data given as wingspan |
| Lepidoptera Callidulidae | 49 | 8 | 14 | 22 | 38 | (Capinera 2008) | Data given as wingspan |
| Lepidoptera Carposinidae | 283 | 3 | 14 | 10 | 40 | (Capinera 2008) | Data given as wingspan |
| Lepidoptera Carthaeidae | 1 | 28 | 37 | 75 | 100 | (Capinera 2008) | Data given as wingspan |
| Lepidoptera Castniidae | 113 | 10 | 82 | 24 | 190 | (Capinera 2008) | Data given as wingspan |
| Lepidoptera Choreutidae | 406 | 3 | 10 | 7 | 24 | (Capinera 2008) | Data given as wingspan |
| Lepidoptera Cimeliidae | 6 | 8 | 11 | 22 | 28 | (Capinera 2008) | Data given as wingspan |
| Lepidoptera Coleophoridae | 1386 | 2 | 7 | 5 | 24 | (Capinera 2008) | Data given as wingspan |
| Lepidoptera Copromorphidae | 43 | 3 | 11 | 12 | 37 | (Capinera 2008) | Data given as wingspan |
| Lepidoptera Cosmopterigidae | 1792 | 2 | 11 | 6 | 32 | (Capinera 2008) | Data given as wingspan |
| Lepidoptera Cossidae | 971 | 5 | 136 | 9 | 240 | (Capinera 2008) | Data given as wingspan |
| Lepidoptera Crinopterygidae | 1 | 2 | 2.5 | 3 | 3.5 | (Kristensen et al. 2007) | Data given as forewing length |
| Lepidoptera Cyclotornidae | 5 | 3 | 10 | 10 | 30 | (Capinera 2008) | Data given as wingspan |
| Lepidoptera Dalceridae | 80 | 4 | 18 | 11 | 50 | (Capinera 2008) | Data given as wingspan |
| Lepidoptera Douglasiidae | 29 | 2 | 5 | 6 | 15 | (Capinera 2008) | Data given as wingspan |
| Lepidoptera Drepanidae | 660 | 6 | 22 | 18 | 66 | (Capinera 2008) | Data given as wingspan |
| Lepidoptera Dudgeoneidae | 57 | 11 | 29 | 28 | 72 | (Capinera 2008) | Data given as wingspan |
| Lepidoptera Elachistidae | 3197 | 2 | 9 | 5 | 23 | (Capinera 2008) | Data given as wingspan |
| Lepidoptera Endromidae | 56 | 11 | 27 | 29 | 74 | (Capinera 2008) | Data given as wingspan |
| Lepidoptera Epicopeiidae | 20 | 13 | 38 | 36 | 126 | (Capinera 2008) | Data given as wingspan |
| Lepidoptera Epipyropidae | 32 | 1 | 13 | 4 | 35 | (Capinera 2008) | Data given as wingspan |
| Lepidoptera Eriocottidae | 80 | 2 | 21 | 5 | 50 | (Capinera 2008) | Data given as wingspan |
| Lepidoptera Eriocraniidae | 28 | 2 | 5 | 6 | 13.5 | (Capinera 2008) | Data given as wingspan |
| Lepidoptera Eupterotidae | 339 | 8 | 47 | 23 | 140 | (Capinera 2008) | Data given as wingspan |
| Lepidoptera Gelechiidae | 4700 | 1 | 12 | 4 | 35 | (Capinera 2008) | Data given as wingspan |
| Lepidoptera Geometridae | 23002 | 3 | 42 | 8 | 120 | (Capinera 2008) | Data given as wingspan |
| Lepidoptera Glyphidoceridae | 49 | 5 | 7 | 13 | 19 | (Capinera 2008) | Data given as wingspan |
| Lepidoptera Glyphipterigidae | 535 | 2 | 14 | 5 | 35 | (Capinera 2008) | Data given as wingspan |
| Lepidoptera Gracillariidae | 1864 | 2 | 10 | 4 | 25 | (Capinera 2008) | Data given as wingspan |
| Lepidoptera Hedylidae | 36 | 15 | 27 | 35 | 65 | (Capinera 2008) | Data given as wingspan |
| Lepidoptera Heliozelidae | 123 | 1 | 3 | 3 | 9 | (Capinera 2008) | Data given as wingspan |
| Lepidoptera Hepialidae | 604 | 8 | 104 | 20 | 250 | (Capinera 2008) | Data given as wingspan |
| Lepidoptera Hesperiidae | 4113 | 7 | 37 | 16 | 82 | (Capinera 2008) | Data given as wingspan |
| Lepidoptera Heterobathmiidae | 3 | 3 | 4 | 10 | 11 | (Capinera 2008) | Data given as wingspan |
| Lepidoptera Heterogynidae | 10 | 3 | 9 | 9 | 29 | (Capinera 2008) | Data given as wingspan |
| Lepidoptera Himantopteridae | 40 | 5 | 12 | 16 | 42 | (Capinera 2008) | Data given as wingspan |
| Lepidoptera Hyblaeidae | 18 | 11 | 22 | 25 | 49 | (Capinera 2008) | Data given as wingspan |
| Lepidoptera Immidae | 245 | 5 | 14 | 14 | 42 | (Capinera 2008) | Data given as wingspan |
| Lepidoptera Incurvariidae | 50 | 2 | 6 | 7 | 18 | (Capinera 2008) | Data given as wingspan |
| Lepidoptera Lacturidae | 120 | 4 | 22 | 11 | 65 | (Capinera 2008) | Data given as wingspan |
| Lepidoptera Lasiocampidae | 1952 | 10 | 92 | 19 | 172 | (Capinera 2008) | Data given as wingspan |
| Lepidoptera Lecithoceridae | 1200 | 2 | 10 | 5 | 30 | (Capinera 2008) | Data given as wingspan |
| Lepidoptera Lemoniidae | 21 | 9 | 28 | 20 | 65 | (Capinera 2008) | Data given as wingspan |
| Lepidoptera Limacodidae | 1672 | 4 | 35 | 9 | 80 | (Capinera 2008) | Data given as wingspan |
| Lepidoptera Lycaenidae | 5201 | 2 | 33 | 6 | 92 | (Capinera 2008) | Data given as wingspan |
| Lepidoptera Lymantriidae | 2500 | 7 | 58 | 16 | 135 | (Capinera 2008) | Data given as wingspan |
| Lepidoptera Lyonetiidae | 220 | 2 | 5 | 4 | 12 | (Capinera 2008) | Data given as wingspan, includes Bedelliidae |
| Lepidoptera Megalopygidae | 232 | 5 | 44 | 10 | 90 | (Capinera 2008) | Data given as wingspan |
| Lepidoptera Micropterigidae | 154 | 1 | 3 | 5 | 12 | (Capinera 2008) | Data given as wingspan |
| Lepidoptera Mimallonidae | 194 | 10 | 28 | 22 | 60 | (Capinera 2008) | Data given as wingspan |
| Lepidoptera Mnesarchaeidae | 7 | 2 | 4 | 5 | 10 | (Capinera 2008) | Data given as wingspan |
| Lepidoptera Momphidae | 115 | 2 | 5 | 8 | 18 | (Capinera 2008) | Data given as wingspan |
| Lepidoptera Neopseustidae | 14 | 3 | 6 | 14 | 27 | (Capinera 2008) | Data given as wingspan |
| Lepidoptera Nepticulidae | 806 | 1 | 2 | 2.5 | 8 | (Capinera 2008) | Data given as wingspan |
| Lepidoptera Noctuidae | 30579 | 3 | 154 | 7 | 360 | (Arnett 2000)/(Ohl & Thiele 2007) | Data given as wingspan |
| Lepidoptera Notodontidae | 3800 | 9 | 57 | 20 | 124 | (Capinera 2008) | Data given as wingspan |
| Lepidoptera Nymphalidae | 6131 | 6 | 56 | 20 | 180 | (Arnett 2000)/(Hogue 1993) | Data given as wingspan |
| Lepidoptera Oecophoridae | 3304 | 2 | 30 | 5 | 80 | (Capinera 2008) | Data given as wingspan |
| Lepidoptera Opostegidae | 192 | 1 | 6 | 3 | 16 | (Capinera 2008) | Data given as wingspan |
| Lepidoptera Palaephatidae | 57 | 3 | 11 | 8 | 36 | (Capinera 2008) | Data given as wingspan |
| Lepidoptera Papilionidae | 566 | 10 | 83 | 35 | 285 | (Capinera 2008) | Data given as wingspan |
| Lepidoptera Pieridae | 1164 | 7 | 31 | 23 | 100 | (Capinera 2008) | Data given as wingspan |
| Lepidoptera Plutellidae | 150 | 2 | 17 | 7 | 55 | (Capinera 2008) | Data given as wingspan |
| Lepidoptera Prodoxidae | 98 | 2 | 11 | 5 | 33 | (Capinera 2008) | Data given as wingspan |
| Lepidoptera Prototheoridae | 12 | 2 | 15 | 6 | 40 | (Capinera 2008) | Data given as wingspan |
| Lepidoptera Psychidae | 1324 | 2 | 37 | 4 | 60 | (Arnett 2000) | Data given as wingspan |
| Lepidoptera Pterolonchidae | 8 | 11 | 12 | 24 | 27 | (Arnett 2000) | Data given as wingspan |
| Lepidoptera Pterophoridae | 1318 | 2 | 15 | 6 | 40 | (Capinera 2008) | Data given as wingspan |
| Lepidoptera Pyralidae | 5921 | 4 | 67 | 5 | 75 | (Resh & Cardé 2009) | Data given as Forewing length, includes Crambidae |
| Lepidoptera Riodinidae | 1532 | 8 | 13 | 20 | 35 | (Arnett 2000) | Data given as wingspan |
| Lepidoptera Roeslerstammiidae | 53 | 4 | 8 | 11 | 22 | (Capinera 2008) | Data given as wingspan |
| Lepidoptera Saturniidae | 2349 | 12 | 117 | 30 | 300 | (Capinera 2008) | Data given as wingspan |
| Lepidoptera Sematuridae | 40 | 19 | 44 | 42 | 100 | (Capinera 2008) | Data given as wingspan |
| Lepidoptera Sesiidae | 1397 | 5 | 34 | 5 | 28 | (Resh & Cardé 2009) | Data given as Forewing length |
| Lepidoptera Somabrachyidae | 8 | 7 | 8 | 18 | 22 | (Capinera 2008) | Data given as wingspan |
| Lepidoptera Sphingidae | 1461 | 11 | 94 | 23 | 200 | (Capinera 2008) | Data given as wingspan |
| Lepidoptera Thyrididae | 940 | 4 | 42 | 9 | 90 | (Capinera 2008) | Data given as wingspan |
| Lepidoptera Tineidae | 2093 | 2 | 21 | 5 | 54 | (Capinera 2008) | Data given as wingspan |
| Lepidoptera Tineodidae | 19 | 7 | 16 | 15 | 34 | (Capinera 2008) | Data given as wingspan |
| Lepidoptera Tischeriidae | 110 | 2 | 3 | 6 | 11 | (Capinera 2008) | Data given as wingspan |
| Lepidoptera Tortricidae | 10387 | 3 | 24 | 7 | 60 | (Capinera 2008) | Data given as wingspan |
| Lepidoptera Uraniidae | 686 | 10 | 50 | 31 | 160 | (Capinera 2008) | Data given as wingspan |
| Lepidoptera Urodidae | 66 | 4 | 14 | 10 | 37 | (Capinera 2008) | Data given as wingspan |
| Lepidoptera Xyloryctidae | 524 | 5 | 29 | 12 | 75 | (Pohl et al. 2010)/(Zborowski & Edwards 2007) | Data given as wingspan |
| Lepidoptera Yponomeutidae | 363 | 2 | 11 | 3.2 | 15 | (Resh & Cardé 2009) | Data given as Forewing length |
| Lepidoptera Ypsolophidae | 163 | 3 | 6 | 9 | 17 | (Capinera 2008) | Data given as wingspan |
| Lepidoptera Zygaenidae | 1036 | 4 | 40 | 5 | 50 | (Resh & Cardé 2009) | Data given as Forewing length |
| Mantodea | 2163 | 10 | 170 |  |  | (Prete 1999) | Represents Order |
| Mantophasmatodea | 16 | 10 | 30 |  |  | (Buder & Klass 2013) |  |
| Mecoptera Apteropanorpidae | 1 | 5.5 | 11 |  |  | (Palmer & Siebke 2008) |  |
| Mecoptera Bittacidae | 214 | 14 | 34 |  |  | (Parker 1982) |  |
| Mecoptera Boreidae | 38 | 2 | 7.5 |  |  | (Parker 1982) |  |
| Mecoptera Choristidae | 12 | 11 | 14 | 13 | 17 | (Riek 1973) | Data as forewing length |
| Mecoptera Meropeidae | 2 | 10 | 12 |  |  | (Arnett 2000) |  |
| Mecoptera Nannochoristidae | 9 | 5 | 9 | 6 | 12 | (Byers 1989) | Data given as wingspan |
| Mecoptera Panorpidae | 480 | 9 | 25 |  |  | (Arnett 2000) |  |
| Mecoptera Panorpodidae | 19 | 7 | 17 |  |  | (Byers 1990) |  |
| Megaloptera Corydalidae | 200 | 20 | 80 |  |  | (Arnett 2000) |  |
| Megaloptera Sialidae | 70 | 13 | 18 |  |  | (Arnett 2000) |  |
| Neuroptera Ascalaphidae | 430 | 40 | 80 |  |  | (Arnett 2000) |  |
| Neuroptera Berothidae | 115 | 6 | 15 | 6 | 15 | (Resh & Cardé 2009) | Data given as Forewing length |
| Neuroptera Chrysopidae | 1200 | 10 | 25 |  |  | (Arnett 2000) |  |
| Neuroptera Coniopterygidae | 450 | 2 | 3 |  |  | (Arnett 2000) |  |
| Neuroptera Hemerobiidae | 550 | 6 | 12 |  |  | (Arnett 2000) |  |
| Neuroptera Ithonidae | 53 | 21 | 40 |  |  | (Arnett 2000) |  |
| Neuroptera Mantispidae | 400 | 20 | 35 |  |  | (Arnett 2000) |  |
| Neuroptera Myrmeleontidae | 2100 | 40 | 80 |  |  | (Arnett 2000) |  |
| Neuroptera Nemopteridae | 100 | 15 | 35 | 15 | 35 | (Resh & Cardé 2009) | Data given as Forewing length |
| Neuroptera Nevrorthidae | 12 | 6 | 10 | 6 | 10 | (Resh & Cardé 2009) | Data given as Forewing length |
| Neuroptera Nymphidae | 35 | 18 | 40 | 18 | 40 | (Resh & Cardé 2009) | Data given as Forewing length |
| Neuroptera Osmylidae | 160 | 15 | 30 | 15 | 30 | (Resh & Cardé 2009) | Data given as Forewing length |
| Neuroptera Polystoechotidae | 4 | 35 | 75 |  |  | (Arnett 2000) |  |
| Neuroptera Psychopsidae | 26 | 10 | 35 | 10 | 35 | (Resh & Cardé 2009) | Data given as Forewing length |
| Neuroptera Sisyridae | 50 | 6 | 8 |  |  | (Arnett 2000) |  |
| Odonata Aeshnidae | 428 | 50 | 100 |  |  | (Garrison et al. 2006) | Data given as wingspan |
| Odonata Austropetaliidae | 11 | 57 | 86 |  |  | (Garrison et al. 2006) |  |
| Odonata Chlorogomphidae | 45 | 60 | 78 |  |  | (Wilson undated) |  |
| Odonata Cordulegastridae | 51 | 55 | 88 |  |  | (Garrison et al. 2006) |  |
| Odonata Corduliidae | 285 | 28 | 68 |  |  | (Garrison et al. 2006) | As subfamily of Libellulidae, Includes Synthemistidae |
| Odonata Gomphidae | 945 | 25.5 | 90 |  |  | (Garrison et al. 2006) |  |
| Odonata Libellulidae | 970 | 17 | 63 |  |  | (Garrison et al. 2006) |  |
| Odonata Macromiidae | 123 | 56 | 91 |  |  | (Garrison et al. 2006) | As subfamily of Libellulidae |
| Odonata Neopetaliidae | 1 | 57 | 58 |  |  | (Garrison et al. 2006) |  |
| Odonata Petaluridae | 11 | 54 | 88 |  |  | (Garrison et al. 2006) |  |
| Odonata Epiophlebiidae | 2 | 48 | 60 |  |  | (Fleck et al. 2013) |  |
| Odonata Calopterygidae | 172 | 45 | 60 |  |  | (Esquivel 1997) |  |
| Odonata Chlorocyphidae | 143 | 26 | 30 |  |  | (Serrano-Meneses et al. 2008) |  |
| Odonata Chorismagrionidae | 1 | 38 | 40 |  |  | (Morton. 1914) |  |
| Odonata Coenagrionidae | 1104 | 16 | 60 |  |  | (Silsby 2001)/(Howarth & Mull 1992) |  |
| Odonata Diphlebiidae | 9 | 45 | 55 |  |  | (Stewart 1980) |  |
| Odonata Euphaeidae | 68 | 26 | 38 |  |  | (Hayashi 1990) |  |
| Odonata Hemiphlebiidae | 1 | 23 | 25 |  |  | (Rivera 2014) |  |
| Odonata Isostictidae | 45 | 15 | 40 |  |  | (Watson 1974) |  |
| Odonata Lestidae | 150 | 40 | 75 |  |  | (Esquivel 1997) |  |
| Odonata Megapodagrionidae | 285 | 40 | 75 |  |  | (Esquivel 1997) |  |
| Odonata Perilestidae | 19 | 50 | 55 |  |  | (Esquivel 1997) |  |
| Odonata Platycnemididae | 222 | 40 | 50 |  |  | (Silsby 2001) |  |
| Odonata Platystictidae | 189 | 40 | 50 |  |  | (Esquivel 1997) |  |
| Odonata Polythoridae | 58 | 30 | 40 |  |  | (Esquivel 1997) |  |
| Odonata Protoneuridae | 240 | 30 | 35 |  |  | (Esquivel 1997) |  |
| Odonata Pseudolestidae | NA | NA | NA |  |  |  |  |
| Odonata Pseudostigmatidae | 19 | 80 | 120 |  |  | (Esquivel 1997) |  |
| Odonata Synlestidae | 33 | 35 | 60 | 50 | 85 | (Picker et al. 2004) | Data given as wingspan |
| Orthoptera Acrididae | 6016 | 9 | 120 |  |  | (Parker 1982) |  |
| Orthoptera Cylindrachetidae | 16 | 35 | 75 |  |  | (Günther 1992)/(Bailey 2007) |  |
| Orthoptera Eumastacoidae | 645 | 10 | 45 |  |  | (Arnett 2000) | Includes Euschmidtiidae, Episactidae, Chorotypidae and Thericleidae |
| Orthoptera Lentulidae | 35 | 12 | 25 |  |  | (Parker 1982) |  |
| Orthoptera Pamphagidae | 448 | 30 | 90 |  |  | (Parker 1982) |  |
| Orthoptera Pneumoridae | 17 | 11.5 | 100 |  |  | (Parker 1982) |  |
| Orthoptera Proscopiidae | 214 | 25 | 165 |  |  | (Parker 1982) |  |
| Orthoptera Pyrgomorphidae | 455 | 10 | 90 |  |  | (Parker 1982) |  |
| Orthoptera Romaleidae | 465 | 18 | 80 |  |  | (Arnett 2000) |  |
| Orthoptera Tanaoceridae | 3 | 10.3 | 25 |  |  | (Parker 1982) |  |
| Orthoptera Tetrigidae | 1246 | 6 | 16 |  |  | (Arnett 2000) |  |
| Orthoptera Tridactylidae | 201 | 4 | 15 |  |  | (Naskrecki 2001) | Includes Rhipipterygidae |
| Orthoptera Trigonopterygidae | 16 | 29 | 40 |  |  | (Ng et al. 2011) |  |
| Orthoptera Xyronotidae | 4 | 17 | 30 |  |  | (Parker 1982) |  |
| Orthoptera Anostostomatidae | 206 | 20 | 80 |  |  | (Pratt et al. 2008) |  |
| Orthoptera Gryllacrididae | 675 | 7 | 50 |  |  | (Arnett 2000) |  |
| Orthoptera Gryllidae | 4664 | 4 | 50 |  |  | (Otte & Alexander 1983) |  |
| Orthoptera Gryllotalpidae | 100 | 20 | 40 |  |  | (Arnett 2000) |  |
| Orthoptera Myrmecophilidae | 8 | 2 | 4 |  |  | (Arnett 2000) |  |
| Orthoptera Prophalangopsidae | 71 | 17 | 30 |  |  | (Walker 2013) |  |
| Orthoptera Rhaphidophoridae | 497 | 10 | 30 |  |  | (Richards 1968)/(Richards 1959) |  |
| Orthoptera Stenopelmatidae | 28 | 30 | 50 |  |  | (Arnett 2000) |  |
| Orthoptera Tettigoniidae | 6827 | 5 | 90 |  |  | (Rentz 2010) |  |
| Phasmatodea Agathemeridae | 8 | 40 | 70 |  |  | (Zompro 2004) |  |
| Phasmatodea Aschiphasmatidae | 96 | 20 | 60 |  |  | (Ng et al. 2011) |  |
| Phasmatodea Bacillidae | 54 | 40 | 110 |  |  | (Scali et al. 2012) /(Picker et al. 2004) |  |
| Phasmatodea Diapheromeridae | 1210 | 17.5 | 140 |  |  | (Zompro 1999)/ (Brock & Hasenpusch 2009) |  |
| Phasmatodea Heteropterygidae | 103 | 20 | 150 |  |  | (Ng et al. 2011) |  |
| Phasmatodea Phasmatidae | 991 | 50 | 357 |  |  | (Ng et al. 2011)/ (Hennemann & Conle 2008) |  |
| Phasmatodea Phylliidae | 51 | 24 | 90 |  |  | (Zompro 2001)/(Ng et al. 2011) |  |
| Phasmatodea Pseudophasmatoidea | 406 | 17.5 | 250 |  |  | (Zompro 1998)/(Picker et al. 2004) | Includes Heteronemiidae |
| Phasmatodea Timematidae | 21 | 12 | 25 |  |  | (Arnett 2000) |  |
| Phthiraptera Boopidae | 55 | 1.3 | 3.14 |  |  | (Parker 1982) |  |
| Phthiraptera Gyropidae | 93 | 0.8 | 1 |  |  | (Parker 1982) |  |
| Phthiraptera Haematomyzidae | 3 | 1.9 | 3 |  |  | (Parker 1982) |  |
| Phthiraptera Heptapsogasteridae | 130 | 0.81 | 4.44 |  |  | (Parker 1982) |  |
| Phthiraptera Laemobothriidae | 20 | 6.5 | 11 |  |  | (Parker 1982) |  |
| Phthiraptera Menoponidae | 1039 | 1.1 | 6 |  |  | (Parker 1982) |  |
| Phthiraptera Philopteridae | 2698 | 1.12 | 9.72 |  |  | (Parker 1982) |  |
| Phthiraptera Ricinidae | 109 | 1.6 | 5.5 |  |  | (Parker 1982) |  |
| Phthiraptera Trichodectidae | 362 | 0.92 | 2.73 |  |  | (Parker 1982) |  |
| Phthiraptera Anoplura | 446 | 0.5 | 5 |  |  | (Arnett 2000) | includes, Echinophthiriidae, Hoplopleuridae, Linognathidae, Pedicinidae, Pediculidae, Pthiridae and Polyplacidae |
| Plecoptera Austroperlidae | 15 | 10 | 35 |  |  | (Parker 1982) |  |
| Plecoptera Capniidae | 287 | 3 | 25 |  |  | (Parker 1982) |  |
| Plecoptera Chloroperlidae | 187 | 6 | 40 |  |  | (Parker 1982) |  |
| Plecoptera Diamphipnoidae | 6 | 25 | 45 |  |  | (Parker 1982) |  |
| Plecoptera Eustheniidae | 23 | 15 | 35 |  |  | (Parker 1982) |  |
| Plecoptera Gripopterygidae | 270 | 5 | 25 |  |  | (Michaelis et al. 2011) |  |
| Plecoptera Leuctridae | 360 | 6 | 13 |  |  | (Arnett 2000) |  |
| Plecoptera Nemouridae | 674 | 6 | 15 |  |  | (Arnett 2000) |  |
| Plecoptera Notonemouridae | 118 | 5 | 8 |  |  | (Picker et al. 2004) |  |
| Plecoptera Peltoperlidae | 69 | 34 | 49 |  |  | (Arnett 2000) |  |
| Plecoptera Perlidae | 965 | 10 | 50 |  |  | (Parker 1982) |  |
| Plecoptera Perlodidae | 310 | 8 | 50 |  |  | (Parker 1982) |  |
| Plecoptera Pteronarcyidae | 12 | 38 | 63 |  |  | (Arnett 2000) |  |
| Plecoptera Scopuridae | 8 | 16 | 25 |  |  | (Jin & Bae 2005) |  |
| Plecoptera Taeniopterygidae | 103 | 10 | 25 |  |  | (Arnett 2000) |  |
| Protura | 712 | 0.6 | 2.5 |  |  | (Arnett 2000) |  |
| Psocoptera Amphientomidae | 100 | 2.3 | 5 |  |  | (New & Lienhard 2007) |  |
| Psocoptera Amphipsocidae | 180 | 2.8 | 5.5 |  |  | (New & Lienhard 2007) |  |
| Psocoptera Archipsocidae | 81 | 1.2 | 1.8 |  |  | (New & Lienhard 2007) |  |
| Psocoptera Caeciliusidae | 566 | 2.5 | 4.5 |  |  | (New & Lienhard 2007) |  |
| Psocoptera Calopsocidae | 34 | 4.3 | 7 |  |  | (New & Lienhard 2007) |  |
| Psocoptera Ectopsocidae | 177 | 2 | 2.5 |  |  | (New & Lienhard 2007) |  |
| Psocoptera Elipsocidae | 129 | 2 | 2.6 |  |  | (New & Lienhard 2007) |  |
| Psocoptera Epipsocidae | 138 | 2.5 | 5.7 |  |  | (New & Lienhard 2007) |  |
| Psocoptera Hemipsocidae | 24 | 2.5 | 2.8 |  |  | (New & Lienhard 2007) |  |
| Psocoptera Lachesillidae | 271 | 1.8 | 2.2 |  |  | (New & Lienhard 2007) |  |
| Psocoptera Lepidopsocidae | 206 | 2 | 2.5 |  |  | (New & Lienhard 2007) |  |
| Psocoptera Liposcelididae | 181 | 1 | 1.5 |  |  | (New & Lienhard 2007) |  |
| Psocoptera Mesopsocidae | 75 | 3.8 | 4.2 |  |  | (New & Lienhard 2007) |  |
| Psocoptera Myopsocidae | 159 | 3 | 5 |  |  | (Arnett 2000) |  |
| Psocoptera Pachytroctidae | 87 | 1.4 | 1.8 |  |  | (New & Lienhard 2007) |  |
| Psocoptera Peripsocidae | 235 | 2 | 4 |  |  | (New & Lienhard 2007) |  |
| Psocoptera Philotarsidae | 111 | 2.2 | 3.8 |  |  | (New & Lienhard 2007) |  |
| Psocoptera Prionoglarididae | 7 | 3 | 3.4 |  |  | (New & Lienhard 2007) |  |
| Psocoptera Pseudocaeciliidae | 899 | 2.5 | 8 |  |  | (New & Lienhard 2007) |  |
| Psocoptera Psilopsocidae | 300 | 1.9 | 3.2 |  |  | (New & Lienhard 2007) |  |
| Psocoptera Psocidae | 7 | 3.2 | 5.4 |  |  | (New & Lienhard 2007) |  |
| Psocoptera Psoquillidae | 27 | 1.1 | 2 |  |  | (New & Lienhard 2007) |  |
| Psocoptera Psyllipsocidae | 26 | 1.3 | 2 |  |  | (New & Lienhard 2007) |  |
| Psocoptera Stenopsocidae | 95 | 3.5 | 4.5 |  |  | (New & Lienhard 2007) |  |
| Psocoptera Trichopsocidae | 11 | 2 | 2.5 |  |  | (Arnett 2000) |  |
| Psocoptera Troctopsocidae | 22 | 1.4 | 4.1 |  |  | (New & Lienhard 2007) |  |
| Psocoptera Trogiidae | 52 | 1.6 | 2.5 |  |  | (New & Lienhard 2007) |  |
| Raphidioptera | 225 | 5 | 20 | 5 | 20 | (Resh & Cardé 2009) | Data as Forewing length |
| Siphonaptera | 2078 | 1 | 10 |  |  | (Whiting et al. 2008) | Represents “Order” |
| Strepsiptera | 590 | 1 | 7.5 |  |  | (Parker 1982) |  |
| Thysanoptera Aeolothripidae | 201 | 1.4 | 2.6 |  |  | (Treherne 1919) |  |
| Thysanoptera Heterothripidae | 76 | 0.6 | 1.5 |  |  | (Retana-Salazar 2009) |  |
| Thysanoptera Phlaeothripidae | 3532 | 2 | 14 |  |  | (Lewis 1973) |  |
| Thysanoptera Thripidae | 2066 | 1 | 3 |  |  | (Arnett 2000) |  |
| Trichoptera Anomalopsychidae | 27 | 4 | 8 |  |  | (Holzenthal & Flint Jr 1995) |  |
| Trichoptera Apataniidae | 203 | 4 | 13 | 8 | 15 | (Ivanov & Menshutkina 1996) | Data as Forewing length |
| Trichoptera Atriplectididae | 6 | 7 | 10 | 20 | 28 | (Neboiss 1986) | Data as wingspan |
| Trichoptera Beraeidae | 57 | 4 | 5 |  |  | (Arnett 2000) |  |
| Trichoptera Brachycentridae | 111 | 6 | 11 |  |  | (Arnett 2000) |  |
| Trichoptera Calamoceratidae | 182 | 6 | 10 | 15 | 26 | (Neboiss 1986) | Data as wingspan |
| Trichoptera Calocidae | 23 | 2 | 10 | 5 | 25 | (Arnett 2000) /(Neboiss 1986) | Data as wingspan |
| Trichoptera Chathamiidae | 5 | 6 | 9 | 15 | 22 | (Neboiss 1986) | Data as wingspan |
| Trichoptera Conoesucidae | 43 | 4 | 10 | 10 | 25 | (Neboiss 1986) | Data as wingspan |
| Trichoptera Dipseudopsidae | 114 | 4 | 14 | 4 | 16 | (Olah & Johanson 2010) | Data as forewing length |
| Trichoptera Ecnomidae | 469 | 2 | 7 | 6 | 18 | (Neboiss 1986) | Data as wingspan |
| Trichoptera Glossosomatidae | 682 | 3 | 10 | 8 | 12 | (Neboiss 1986) | Data as wingspan |
| Trichoptera Goeridae | 184 | 4 | 10 | 5 | 12 | (Parker 1998)/(Greenhalgh & Ovenden 2004) | Data as forewing length |
| Trichoptera Helicophidae | 44 | 3 | 6 | 8 | 15 | (Neboiss 1986) | Data as wingspan |
| Trichoptera Helicopsychidae | 269 | 4 | 6 | 10 | 16 | (Neboiss 1986) | Data as wingspan |
| Trichoptera Hydrobiosidae | 407 | 4 | 13 | 10 | 35 | (Neboiss 1986) | Data as wingspan |
| Trichoptera Hydropsychidae | 1808 | 3 | 21 | 8 | 56 | (Neboiss 1986) /(Picker et al. 2004) | Data as wingspan |
| Trichoptera Hydroptilidae | 2124 | 1.5 | 4 | 4 | 12 | (Neboiss 1986) | Data as wingspan |
| Trichoptera Kokiriidae | 15 | 5 | 9 | 14 | 24 | (Neboiss 1986) | Data as wingspan |
| Trichoptera Lepidostomatidae | 471 | 8 | 10 |  |  | (Arnett 2000) |  |
| Trichoptera Leptoceridae | 2020 | 4 | 15 | 10 | 40 | (Neboiss 1986) | Data as wingspan |
| Trichoptera Limnephilidae | 880 | 7 | 23 | 25 | 40 | (Neboiss 1986) | Data as wingspan |
| Trichoptera Limnocentropodidae | 15 | 10 | 12 | 27 | 33 | (Wiggins 1956) | Synonym Kitagamiidae, Data as wingspan |
| Trichoptera Molannidae | 41 | 10 | 17 |  |  | (Arnett 2000) |  |
| Trichoptera Odontoceridae | 154 | 5 | 14 | 14 | - | (Neboiss 1986)/(Arnett 2000) | Minimum as wingspan/ body length |
| Trichoptera Oeconesidae | 18 | 12 | 16 | 30 | 40 | (Neboiss 1986) | Data as wingspan |
| Trichoptera Philopotamidae | 1168 | 6 | 9 | 12 | 20 | (Neboiss 1986) | Data as wingspan |
| Trichoptera Philorheithridae | 30 | 6 | 13 | 16 | 35 | (Neboiss 1986) | Data as wingspan |
| Trichoptera Phryganeidae | 84 | 12 | 28 | 18 | 43 | (Wiggins 1998) | Data as forewing length |
| Trichoptera Pisuliidae | 19 | 6 | 19 | - | 40 | (Morse 1974)/ (Picker et al. 2004) | Maximum as wingspan |
| Trichoptera Polycentropodidae | 806 | 5 | 10 | 8 | 25 | (Neboiss 1986) | Data as wingspan |
| Trichoptera Psychomyiidae | 522 | 4 | 6 |  |  | (Arnett 2000) |  |
| Trichoptera Rhyacophilidae | 774 | 8 | 13 |  |  | (Arnett 2000) |  |
| Trichoptera Sericostomatidae | 107 | 8 | 14 | 20 | 35 | (Picker et al. 2004) | Data as wingspan |
| Trichoptera Stenopsychidae | 94 | 6 | 12 | 18 | 35 | (Neboiss 1986) | Data as wingspan |
| Trichoptera Tasimiidae | 9 | 4 | 6 | 12 | 18 | (Neboiss 1986) | Data as wingspan |
| Trichoptera Uenoidae | 31 | 7 | 9 | 8 | 10 | (Houghton 2012) | Data given as forewing length |
| Trichoptera Xiphocentronidae | 172 | 3 | 4 | 3 | 4 | (Munoz-Quesada & Holzenthal 1997) | Data given as forewing length |
| Zoraptera | 35 | 2 | 3 |  |  | (Parker 1982) |  |
| Zygentoma Lepidotrichidae | 1 | 12 | 14 |  |  | (Arnett 2000)/(Resh & Cardé 2009) |  |
| Zygentoma Lepismatidae | 200 | 8 | 20 |  |  | (Arnett 2000) |  |
| Zygentoma Nicoletiidae | 30 | 4 | 29 |  |  | (Arnett 2000)/(Espinasa et al. 2013) |  |

Table S2. Outputs of Macrocaic analysis of relationship between PIC of diversification rate (measured as PDI) and mean log size for major clades. See text for discussion.

| Taxa | N (Contrasts) | Estimate | (Adj) R^2^ | SE | t | p |
| --- | --- | --- | --- | --- | --- | --- |
| Hexapoda | 773 | 0.4589 | 0.002572 | 0.2652 | 1.73 | 0.084 |
| Holometabola | 507 | 0.5020 | 0.003065 | 0.3138 | 1.6 | 0.11 |
| Paraneoptera | 126 | 1.231 | 0.01155 | 0.783 | 1.573 | 0.118 |
| Polyneoptera | 64 | 0.6955 | -0.007437 | 0.9576 | 0.726 | 0.47 |
| Palaeoptera | 57 | -1.524 | 0.01207 | 1.170 | -1.303 | 0.198 |
| Ectognatha | 11 | 0.9674 | -0.07041 | 1.8400 | 0.526 | 0.611 |

Table S3. Parameter estimates and relative likelihoods of alternative models of mean body size for major orders of Holometabola (including terminal standard error). Models and parameters denoted as in Table 3.

| Clade | Model | Sigma squared | z0 | a/delta/alpha | LnLik | k | AICc | Delta AiCc from optimal model | Akaike weights |
| --- | --- | --- | --- | --- | --- | --- | --- | --- | --- |
| Hymenoptera | **BM** | **0.003168** | **2.091** |  | **-86.87** | **2** | **177.9** | **0** | **0.4210** |
|  | EB | 0.003952 | 2.105 | -0.001230 | -86.81 | 3 | 179.9 | 2.043 | 0.1516 |
|  | delta | 0.003159 | 2.090 | 1.006 | -86.87 | 3 | 180.1 | 2.166 | 0.1425 |
|  | SSP | 0.003168 | 2.091 | 0.000 | -86.87 | 3 | 180.1 | 2.167 | 0.1425 |
|  | lambda | 0.003168 | 2.091 | 1 | -86.87 | 3 | 180.1 | 2.167 | 0.1425 |
|  | WN | 0.8712 | 1.784 |  | -104.1 | 2 | 212.3 | 34.39 | 0.0000 |
|  |  |  |  |  |  |  |  |  |  |
| Diptera | BM | 0.003120 | 1.635 |  | -114.8 | 2 | 233.7 | 16.94 | 0.00014 |
|  | EB | 0.003121 | 1.635 | -1e-06* | -114.8 | 3 | 235.8 | 19.04 | 0.00005 |
|  | delta | 0.001396 | 1.539 | 4.392 | -106.8 | 3 | 219.9 | 3.117 | 0.1357 |
|  | SSP | 0.006735 | 1.550 | 0.007896 | -106.4 | 3 | 219.0 | 2.195 | 0.2152 |
|  | **lambda** | **0.001695** | **1.611** | **0.6648** | **-105.3** | **3** | **216.7** | **0** | **0.6449** |
|  | WN | 0.3991 | 1.513 |  | -111.4 | 2 | 227.0 | 10.21 | 0.0039 |
|  |  |  |  |  |  |  |  |  |  |
| Coleoptera | **BM** | **0.002685** | **1.424** |  | **-153.6** | **2** | **311.3** | **0.5992** | **0.2071** |
|  | EB | 0.002686 | 1.424 | -1e-06* | -153.6 | 3 | 313.4 | 2.689 | 0.0729 |
|  | delta | 0.002091 | 1.494 | 1.656 | -152.34 | 3 | 310.9 | 0.1922 | 0.2538 |
|  | SSP | 0.003932 | 1.467 | 0.002282 | -152.3 | 3 | 310.7 | **0** | 0.2794 |
|  | lambda | 0.00228 | 1.436 | 0.8274 | -152.7 |  | 311.5 | 0.8054 | 0.1868 |
|  | WN | 0.5859 | 1.625 |  | -162.6 | 2 | 329.2 | 18.47 | 0.00003 |
|  |  |  |  |  |  |  |  |  |  |
| Lepidoptera | **BM** | **0.002756** | **1.368** |  | **-95.14** | **2** | **194.4** | **1.661** | **0.1996** |
|  | EB | 0.002756 | 1.368 | -1e-06* | -95.14 | 3 | 196.5 | 3.778 | 0.0692 |
|  | delta | 0.002012 | 1.488 | 1.618 | -94.46 | 3 | 195.2 | 2.415 | 0.1369 |
|  | SSP | 0.003441 | 1.444 | 0.001989 | -94.47 | 3 | 195.2 | 2.420 | 0.1365 |
|  | lambda | 0.002197 | 1.393 | 0.88127 | -93.26 | 3 | 192.7 | 0 | 0.4578 |
|  | WN | 0.5985 | 2.106 |  | -125.7 | 2 | 255.4 | 62.68 | 0.0000 |

**Supplementary References**

**Size data**

Arnett RH: American insects: A handbook of the insects of America north of Mexico. Boca Raton, Fla.: CRC Press; 2000.

Arnett RH, Thomas, MC, editors: American beetles, Volume I: Archostemata, Myxophaga, Adephaga, Polyphaga: Staphyliniformia. Boca Raton, Fla.: CRC Press; 2000.

Arnett RH, Thomas MC, Skelley PE, Frank JH, editors. American beetles, Volume II: Polyphaga: Scarabaeoidea through Curculionoidea. Boca Raton, Fla.: CRC Press; 2010.

Bae Y, McCafferty WP. Phylogenetic systematics and biogeography of the Neoephemeridae (Ephemeroptera: Pannota). Aquatic Insects 1998, 20:35–68.

Bailey PT. Pests of Field Crops and Pastures: Identification and Control. Collingwood: CSIRO Publishing; 2007.

Barnes JK. Revision of the Helosciomyzidae (Diptera). J Roy Soc New Zealand 1981, 11:45–72.

Bechev D, Chandler P. Catalogue of the Bolitophilidae and Diadocidiidae of the world (Insecta: Diptera). Zootaxa 2011 , 2741:38–58.

Berenbaum M. Lend me your earwigs. Amer Entomol 2007, 53:196–197.

Beutel RG, Leschen R, editors. Handbook of zoology. Volume IV. Arthropoda: Insecta. Part 38. Coleoptera. Beetles. Volume 1: Morphology and Systematics (Archostemata, Adephaga, Myxophaga, Polyphaga partim). Berlin: Walter de Gruyter; 2005.

Boeseman M. The Dermaptera in the museums at Leiden and Amsterdam. Leiden: Brill Academic Publishing; 1954.

Bouček Z, Noyes JS. Rotoitidae, a curious new family of Chalcidoidea (Hymenoptera) from New Zealand. Syst Entomol 1987, 12:407–412.

Brake I, Mathis WN. Revision of the genus *Australimyza* Harrison (Diptera: Australimyzidae). Syst Entomol 2007, 32:252–275.

Brock PD, Hasenpusch JW. The complete field guide to stick and leaf insects of Australia. Collingwood: CSIRO Publishing; 2009.

Buder G, Klass K-D. The morphology of tarsal processes in Mantophasmatodea. Deutsche Entomol Zeitschr 2013, 60:5–23.

Byers GW. The Nannochoristidae of South America (Mecoptera). Univ Kans Sci Bull 1989, 54:25–34.

Byers GW. *Brachypanorpa sacajawea* n. sp. (Mecoptera: Panorpodidae) from the Rocky Mountains. J Kansas Entomol Soc 1990, 63:211–217.

Cambra R, Oliveira A. First Central American record of *Clystopsenella longiventris* (Hymenoptera: Scolebythidae) with comments on the variation of the species. Entomotropica 2003, 18:147–148.

Capinera JL, editor. Encyclopedia of entomology, 2nd Edition. Dordrecht: Springer; 2008.

Colless D. The genus *Perissomma* (Diptera : Perissommatidae) with new species from Australia and Chile. Australian J Zool 1969, 17:719–728.

Darilmaz MC, Kiyak S. A study of the family Spercheidae (Coleoptera) from Turkey. Turk J Zool 2011, 35:441–444.

Deitz LL, Wallace MS (team leaders): Treehoppers: Aetalionidae, Melizoderidae, and Membracidae (Hemiptera). 2010. http://treehoppers.insectmuseum.org. Accessed 1st Oct 2012.

Doganler M. Notes on the species of Tetracampidae with descriptions of some new species from Turkey. Entomofauna 2003, 24:381–396.

Domínguez E, Adis J, Arias JR; Hubbard MD; Molineri C; Nieto C et al. Ephemeroptera de América Del Sur. Sofia: Pensoft Publishers; 2006.

Early JW, Masner L, Naumann ID, Austin AD. Maamingidae, a new family of proctotrupoid wasp (Insecta : Hymenoptera) from New Zealand. Invert Syst 2001, 15:341–352.

Edmunds GF, Jensen SL, Berner L. The mayflies of North and Central America. Minneapolis: University of Minnesota Press; 1976.

Espinasa L, Botelho M, Socci K. A new species of genus *Squamigera* (Insecta: Zygentoma: Nicoletiidae) from the Mayan ruins of Palenque, Chiapas, Mexico. J Entomol Nematol 2013, 52:24–28.

Esquivel C. The families of insects of Costa Rica- Odonata. In Solís A, editor, Las familias de insectos de Costa Rica. 1997.http://www.inbio.ac.cr/papers/insectoscr/Texto228.html. Accessed 1st Jan 2014.

Evans JW. The leafhoppers and froghoppers of Australia and New Zealand (Homoptera: Cicadelloidea and Cercopoidea). Australian Museum Memoir 1966, 12:1–347.

Evenhuis NL. Family Xenasteiidae. In: Evenhuis NL, editor. Catalog of the Diptera of the Australasian and Oceanian Regions. 2011. http://hbs.bishopmuseum.org/aocat/hybotidae.html. Accessed 1st Jan 2013.

Fleck G, Li J, Schorr M, Nel A, Zhang X, Lin L, Gao M. *Epiophlebia sinensis* Li & Nel 2011 in Li et al. (2012) (Odonata) newly recorded in North Korea. International Dragonfly Fund Report 2013, 61:1–4.

Foottit RG, Adler PH. Insect biodiversity: science and society. Oxford: John Wiley & Sons; 2009.

Garrison RW, von Ellenrieder N, Louton JA. Dragonfly genera of the New World: an illustrated and annotated key to the Anisoptera. Baltimore: Johns Hopkins University Press; 2006.

Gibson GAP, Read J, Huber JT. Diversity, classification and higher relationships of Mymarommatoidea (Hymenoptera). J Hym Res 2007, 16:51-146.

Gillies MT. The african Euthyplociidae (Ephemeroptera), (Exeuthyplociinae subfam. n.). Aquatic Insects 1980, 2:217–224.

Greenhalgh M, Ovenden D. The flyfisher’s handbook. Machynleth: Coch Y Bonddu Books; 2004.

Günther KK. Revision der Familie Cylindrachetidae Giglio-Tos, 1914 (Orthoptera, Tridactyloidea). Deutsche Entomol Zeitschr 1992, 39:233–291.

Hayashi F. Convergence of insular dwarfism in damselflies (*Euphaea*) and dobsonflies (*Protohermes*). Freshw Biol 1990, 23:219–231.

Heckman CW. Encyclopedia of South American aquatic insects: Ephemeroptera: illustrated keys to known families, genera, and species in South America. Dordrecht: Springer; 2002.

Hennemann FH, Conle OV. Revision of Oriental Phasmatodea: The tribe Pharnaciini Günther, 1953, including the description of the world’s longest insect, and a survey of the family Phasmatidae Gray, 1835 with keys to the subfamilies and tribes (Phasmatodea: “Anareolatae”: Phasmatidae). Zootaxa 2008, 1906:1–316.

Hisamatsu S. A review of the Japanese Kateretidae fauna (Coleoptera: Cucujoidea). Acta Entomologica Musei Nationalis Pragae 2011, 51:551–585.

Hitchings TR, Staniczek AH. Nesameletidae (Insecta: Ephemeroptera). Fauna of New Zealand 2003, 46:1-72.

Hogue CL. Latin american insects and entomology. Berkeley: University of California Press; 1993.

Holzenthal RW, Flint OS Jr. Studies of Neotropical caddisflies, LI: systematics of the Neotropical caddisfly genus *Contulma* (Trichoptera: Anomalopsychidae). Smithsonian Contributions to Zoology 1995, 575:1-59

Hopkin SP. Biology of the springtails : (Insecta: Collembola). Oxford: Oxford University Press; 1997.

Houghton DC. Biological diversity of the Minnesota caddisflies (Insecta, Trichoptera). ZooKeys 2012, 189:1–389.

Houston TF. A revision of the bee genus *Ctenocolletes* (Hymenoptera: Stenotritidae). Records of the Western Australian Museum 1983, 10:269–306.

Howarth FG, Mull WP. Hawaiian insects and their kin. Honolulu: University of Hawaii Press; 1992.

Ivanov VD, Menshutkina T. Endemic Caddisflies of Lake Baikal (Trichoptera Apataniidae). Braueria 1996, 23:13–28.

Jäch MA, Balke M. Key to the adults of Chinese water beetle families. In Jäch MA, Ji L, editors. Water beetles of China Vol III. Wien: Zoologisch-Botanische Gesellschaft in Österreich and Wiener Coleopterologenverein. 2003. p. 21–36.

Jin Y, Bae Y. The wingless stonefly family Scopuridae (Plecoptera) in Korea. Aquatic Insects 2005, 27:21–34.

Karpa A. Revision of the Chloropidae of the collection of B.A. Gimmerthal and a check list of Latvian Chloropidae (Diptera). Latvijas Entomologs 2001, 38:44–49.

Kristensen NP, Scoble M, Karsholt O. Lepidoptera phylogeny and systematics: the state of inventorying moth and butterfly diversity. Zootaxa 2007, 1668:699–747.

Lenhart P, Dash ST, Mackay WP. A revision of the giant Amazonian ants of the genus *Dinoponera* (Hymenoptera, Formicidae). J Hym Res 2013, 31:119–164.

Leschen RAB, Beutel RG, Lawrence JF. Handbook of zoology. Arthropoda: Insecta. Coleoptera. Beetles. Volume 2: Morphology and Systematics (Elateroidea, Bostrichiformia, Cucujiformia partim). Berlin: Walter de Gruyter; 2010.

Lewis T. Thrips: their biology, ecology and economic importance. London: Academic Press; 1973.

Marsh N. Trout stream insects of New Zealand. Aukland: The Halcyon Books; 2004.

Mathis WN. World catalog and conspectus on the family Helcomyzidae (Diptera: Schizophora). Myia 2011, 12:267–280.

Mathis WN. World catalog and conspectus on the family Heterocheilidae (Diptera: Schizophora). Myia 2011, 12:281–289.

Mathis WN, Sueyoshi M. World catalog and conspectus on the family Dryomyzidae. Myia 2011, 12: 207–233.

McAlpine DK. Marginidae a new afrotropical family of Diptera (Schizophora: ? Opomyzoidea). Annals of the Natal Museum 1991, 32:167–178.

McAlpine DK. Review of the Upside-down Flies (Diptera: Neurochaetidae) of Madagascar and Africa, and evolution of neurochaetid host plant associations. Records of the Australian Museum 1993, 45:221–239.

McAlpine JF, Peterson BV, Shewell GE, Teskey HJ, Vockeroth JR, Wood DM, editors. Manual of nearctic Diptera. Volume 1. Research Branch, Agriculture Canada; 1981.

McAlpine JF, Peterson BV, Shewell GE, Teskey HJ, Vockeroth JR, Wood DM, editors. Manual of nearctic Diptera. Volume 2. Research Branch, Agriculture Canada; 1987.

Mercado M, Elliot S. Taxonomic revision of the genus *Chiloporter* Lestage (Ameletopsidae: Ephemeroptera) with notes on its biology and distribution. Studies on Neotropical Fauna and Environment 2005, 40:229–236.

Messer AC. *Chalicodoma pluto*: the world’s largest bee rediscovered living communally in termite nests (Hymenoptera: Megachilidae). J Kansas Entomol Soc 1984, 57:165–168.

Michaelis FB, Yule C, Calder A. Family Gripopterygidae. In: Australian Faunal Directory. Australian Biological Resources Study, Canberra. 2011. http://archive.is/Ckwa#selection-249.0-249.22. Accessed 1st Jan 2014.

Morse JC. New caddisflies (Trichoptera) from Southern Africa. J Kansas Entomol Soc 1974, 47:328–344.

Morton KJ. IV. A remarkable new genus and new species of Odonata, of the legion *Podagrion*, Selys, from North Queensland. Transactions of the Royal Entomological Society of London 1914, 62:169–172.

Munoz-Quesada F, Holzenthal RW. A new species of *Xiphoncentron* (*Antillotrichia*) from Costa Rica with semiterrestrial immature stages (Trichoptera Xiphoncentronidae). Proceedings of the 8th International Symposium on Trichoptera 1997:355–363.

Nagatomi A, Nagatomi H. The genus *Austroleptis* from South Chile and Patagonia (Diptera, Rhagionidae). Memoirs of the Kagoshima University Research Center for the South Pacific 1987, 8:139–156.

Nagatomi A, Saigusa T, Nagatomi H, Lynebord L. Apsilocephalidae, a new family of orthorrhaphous Brachycera (Insecta, Diptera). Zoological Science (Tokyo) 1991, 8:579–591.

Naskrecki P. 2001. Grasshoppers and their relatives. In: Levine SA, editor. Encyclopedia of biodiversity 3. San Diego: Academic Press; 2001. p. 247-264.

Neboiss A. Atlas of Trichoptera of the SW Pacific-Australian region. Dordrecht: W. Junk; 1986.

Nelson LA, Scheffer SJ, Yeates DK. Two new species of sympatric *Fergusonina* Malloch flies (Diptera: Fergusoninidae) from bud galls on high-elevation snow gums (*Eucalyptus pauciflora* Sieb. ex Spreng. complex) in the Australian Alps. Australian J Entomol 2011, 50:356–364.

New T, Lienhard C. The Psocoptera of tropical South East Asia. Leiden: Brill Academic Publishing; 2007.

Ng PKL, Corlett R, Tan HTW. Singapore biodiversity: an encyclopedia of the natural environment and sustainable development. Singapore: Editions Didier Millet; 2011.

Ohl M, Thiele K. Estimating body size in apoid wasps: the significance of linear variables in a morphologically diverse taxon (Hymenoptera, Apoidea). Zoosystematics and Evolution 2007, 83:110–124.

Olah J, Johanson KA. Contributions to the systematics of the genera *Dipseudopsis* , *Hyalopsyche* and *Pseudoneureclipsis* (Trichoptera: Dipseudopsidae), with descriptions of 19 new species from the Oriental Region. Zootaxa 2010, 2658:1–37.

Oosterbroek P. The families of Diptera of the malay archipelago. Leiden: Brill Academic Publishing; 1998.

Otte D, Alexander RD. Australian crickets (Orthoptera: Gryllidae). Philadelphia: Academy of Natural Sciences of Philadelphia; 1983.

Palaczyk A, Klasa A, Slowinska-Krysiak I. The family Strongylophthalmyiidae of Poland with catalog of European species (Insecta: Diptera). Genus 2013, 24:425–438.

Palmer CM, Siebke K. Cold hardiness of *Apteropanorpa tasmanica* Carpenter (Mecoptera: Apteropanorpidae). J Insect Physiol 2008, 54:1148–1156.

Papp L. A study on *Hesperinus* (Walker) with description of a new species (Diptera: Hesperinidae). Acta Zoologica Academiae Scientiarum Hungaricae 2010, 56:347–370.

Parker C. A review of *Goerita* (Trichoptera: Goeridae), with description of a new species. Insecta Mundi 1998, 12: 227-238.

Parker SP. Synopsis and classification of living organisms. New York: Mc-Graw Hill Book Company, 1982.

Pearson RG, Penridge LK. First records of *Prosopistoma sedlaceki* in Australia (Ephemeroptera Prosopistomatidae). J Australian Entomol Soc 1979, 18:362.

Penniket JG. Notes on New Zealand Ephemeroptera III. A new family genus and species. Records of the Canterbury Museum 1962, 7:389–398.

Penniket JG. Notes on New Zealand Ephemeroptera IV. A new Siphlonurid subfamily; Rallidentinae. Records of the Canterbury Museum 1966, 8:163–175.

Phillips JS. A revision of New Zealand Ephemeroptera Part 1. Transactions and Proceedings of the Royal Society of New Zealand 1930, 61:271–334.

Picker M, Griffiths C, Weaving A. field guide to insects of South Africa. Cape Town: Struik Nature, 2004.

Pohl G, Anweiler GC, Schmidt BC, Kondla NG. Annotated list of the Lepidoptera of Alberta, Canada. Zookeys2010, 38:1-549.

Pratt RC, Morgan-Richards M, Trewick SA. Diversification of New Zealand weta (Orthoptera: Ensifera: Anostostomatidae) and their relationships in Australasia. Phil Trans Roy Soc B 2008, 363:3427–3437.

Prete FR. The praying mantids. Baltimore: Johns Hopkins University Press; 1999.

Rentz D. A guide to the katydids of Australia. Collingwood: CSIRO Publishing; 2010.

Resh VH, Cardé RT. Encyclopedia of insects, Second Edition. San Diego: Elsevier; 2009.

Retana-Salazar A. Species of *Heterothrips* Hood (Terebrantia: Heterothripidae) from Central America. Ceiba 2009, 50:10–17.

Richards A. Revision of the Rhaphidophoridae (Orthopera) of New Zealand Part IV: The Raphidophoridae of Thames Gold Mines. Trans Roy Soc New Zealand 1959, 87:27–33.

Richards A. The Raphidophoridae (Orthopera) or Australia: Part 6 Two New Species from Northern Tasmania. Pacific Insects 1968, 10:167–176.

Riek EF. A Revision of Australian scorpion flies of the family Choristidae (Mecoptera). Australian J Entomol 1973, 12:103–112.

Rivera AC. Behaviour and ecology of *Hemiphlebia mirabilis* (Odonata: Hemiphlebiidae). 2014. http://natureglenelg.org.au/wp-content/uploads/2014/02/Behaviour-and-ecology-of-Hemiphlebia-mirabilis-by-Adolfo-Cordero-Rivera-2014.pdf. Accessed 1st July 2014.

Robinson WH. Urban insects and arachnids: a handbook of urban entomology. Cmabridge: Cambridge University Press; 2005.

Scali V, Milani L, Passmonti M. Revision of the stick insect genus *Leptynia* : description of new taxa, speciation mechanism and phylogeography. Contributions to Zoology 2012, 81:25–42.

Schneeberg K, Krause K, Beutel RG. The adult head of *Axymyia furcata* (Insecta: Diptera: Axymyiidae). Arthropod Systematics & Phylogeny 2013, 71:91–102.

Schuh RT, Slater JA. True bugs of the world (Hemiptera:Heteroptera): classification and natural history. Ithaca: Cornell University Press; 1995.

Serrano-Meneses MA, Córdoba-Aguilar A, Azpilicueta-Amorín M, González-Soriano E, Székely T. Sexual selection, sexual size dimorphism and Rensch’s rule in Odonata. J Evol Biol 2008, 21:1259–1273.

Shockley FW. Alexiidae Imhoff 1856. *Sphaerosoma* Samouelle 1819. 2008. http://tolweb.org/Sphaerosoma/65850/2008.06.2. Accessed 1st July 2014.

Shockley FW, Hartley CS, Lord N. Latridiidae. Minute brown scavenger beetles. 2011. http://tolweb.org/Latridiidae/9172/2011.03.23. Accessed 1st July 2014.

Silsby J. Dragonflies of the world. Collingwood: CSIRO Publishing; 2001.

Stewart W. The Australian genus *Diphlebia* Selys (Odonata : Amphipterygidae). I. Taxonomic revision of the adults. Australian J Zoology Supplementary Series 1980, 28:1–57.

Tojo K, Matsukawa K. A description of the second species of the family Dipteromimidae (Insecta, Ephemeroptera), and genetic relationship of two Dipteromimid mayflies inferred from mitochondrial 16S rRNA gene sequences. Zoological Science 2003, 20:1249–1259.

Treherne RC. Notes on the Aeolothripidae. J Entomol Soc British Columbia 1919, 12:27–33.

Walker T. Family Prophalangopsidae (hump-winged grigs) in North America north of Mexico. 2013. In Encyclopedia of life. Available at: http://eol.org/pages/991/overview. Accessed 1st July 2014.

Wall R, Shearer D. Veterinary ectoparasites: biology, pathology and control. Oxford: John Wiley & Sons; 2008.

Watson JAL. The distributions of the Australian dragonflies (Odonata). J Australian Entomol Soc 1974, 13:137–149.

Whiting MF, Whiting AS, Hastriter MW, Dittmar K. A molecular phylogeny of fleas (Insecta: Siphonaptera): origins and host associations. Cladistics 2008, 24: 677–707.

Wiegmann BM. A phylogenetic revision of the family Atelestidae (Diptera: Empidoidea) and its implications for the origin of the Cyclorrhaphous Diptera, MSc Thesis

Wiggins GB. The Kitagamiidae, a family of caddisflies new to North America (Trichoptera). Contributions of the Royal Ontario Museum of Zoology and Palaeontology 1956, 44:1-10. s, University of Maryland; 1989.

Wiggins GB. The caddisfly family Phryganeidae (Trichoptera). Toronto: University of Toronto Press; 1998.

Wilson KD. Dragonfly groups of conservation interest confined to the Oriental Region. [https://www.yumpu.com/en/document/view/39638885/dragonfly-groups-of-conservation-interest-confined-asia-dragonfly Accessed 1st July 2014](https://www.yumpu.com/en/document/view/39638885/dragonfly-groups-of-conservation-interest-confined-asia-dragonfly%20Accessed%201st%20July%202014).

Zborowski P, Edwards T. A guide to australian moths. Collingwood: CSIRO Publishing; 2007.

Zloty J, Pritchard G. Larvae and adults of *Ameletus* mayflies (Ephemeroptera Ameletidae) from Alberta. Can Entomol 1997, 129:251–289.

Zompro O. New phasmids from Venezuela and Ecuador Phasmatodea Neue Phasmiden aus Venezuela und Ecuador Phasmatodea. Entomologische Zeitschrift 1998, 10811:456–459.

Zompro O. *Microphasma*, a new stick insect genus from Sri Lanka. (Phasmatodea: Pachymorphinae). Entomological Journal 1999, 109:124–127.

Zompro O. Philippine phasmids from the collection of the Staatliches Museums für Tierkunde, Dresden (Insecta: Phasmatodea). Reichenbachia 2001, 34:49-56.

Zompro O. Revision of the genera of the Areolatae, including the status of *Timema* and *Agathemera* (Insecta, Phasmatodea). Keltern-Weiler; Goecke & Evers; 2004.

R packages

R language: R Development Core Team. 2011. R: A Language and Environment for Statistical Computing. R Foundation for Statistical Computing.

*ape* : Paradis, E., Claude, J. & Strimmer, K. 2004. APE: Analyses of Phylogenetics and Evolution in R language. *Bioinformatics* 20: 289–290.

*BAMMtool*s: Rabosky, D.L., Grundler, M., Anderson, C., Title, P., Shi, J.J., Brown, J.W., *et al.* 2014. BAMMtools: an R package for the analysis of evolutionary dynamics on phylogenetic trees. *Methods Ecol. Evol.* 5: 701–707.

*caper* : Orme, C.D.L., Freckleton, R.P., Thomas, G.H., Petzoldt, T., Fritz, S.A., Isaac, N.J.B., *et al.* 2012. caper: Comparative Analyses of Phylogenetics and Evolution in R.

*geiger*: Harmon, L.J., Weir, J.T., Brock, C.D., Glor, R.E. & Challenger, W. 2008. GEIGER: investigating evolutionary radiations. *Bioinformatics* 24: 129–131. / Pennell, M.W., Eastman, J.M., Slater, G.J., Brown, J.W., Uyeda, J.C., FitzJohn, R.G., *et al.* 2014. geiger v2.0: an expanded suite of methods for fitting macroevolutionary models to phylogenetic trees. *Bioinformatics* 30: 2216–2218.

*moments*: Komsta, L. & Novomestky, F. 2012. moments: Moments, cumulants, skewness, kurtosis and related tests. R package version 0.13.

*phytools*: Revell, L.J. 2012. phytools: an R package for phylogenetic comparative biology (and other things). *Methods Ecol. Evol.* 3: 217–223.
